# Mental health impacts of COVID-19: A retrospective analysis of dynamic modelling projections for Australia

**DOI:** 10.1016/j.heliyon.2024.e28250

**Published:** 2024-03-16

**Authors:** Adam Skinner, Jo-An Occhipinti, Yun Ju Christine Song, Ian B. Hickie

**Affiliations:** a Brain and Mind Centre, Faculty of Medicine and Health, University of Sydney, Sydney, Australia; b Computer Simulation and Advanced Research Technologies (CSART), Sydney, Australia

**Keywords:** Pandemic, Psychological distress, Social disconnection, Suicide, System dynamics, Unemployment

## Abstract

**Background:**

In early 2020, we developed a dynamic model to support policy responses aimed at mitigating the adverse mental health effects of the COVID-19 pandemic in Australia. As the pandemic has progressed, it has become clear that our initial model forecasts overestimated the impacts of infection control measures (lockdowns, physical distancing, etc.) on suicide, intentional self-harm hospitalisation, and mental health-related emergency department (ED) presentation rates.

**Methods:**

Potential explanations for the divergence of our model predictions from observed outcomes were assessed by comparing simulation results for a set of progressively more refined models with data on the prevalence of moderate to very high psychological distress and numbers of suicides, intentional self-harm hospitalisations, and mental health-related ED presentations published after our modelling was released in July 2020.

**Results:**

Allowing per capita rates of spontaneous recovery and intentional self-harm to differ between people experiencing moderate to very high psychological distress prior to the pandemic and those developing comparable levels of psychological distress only as a consequence of infection control measures substantially improves the fit of our model to empirical estimates of the prevalence of psychological distress and leads to significantly lower predicted effects of COVID-19 on suicide, intentional self-harm hospitalisation, and mental health-related ED presentation rates.

**Conclusion:**

Accommodating the influence of prior mental health on the psychological effects of population-wide social and economic disruption is likely to be critical for accurately forecasting the mental health impacts of future public health crises as they inevitably arise.

## 1 Introduction

During the first half of 2020, shortly after the World Health Organization declared the coronavirus disease 2019 (COVID-19) outbreak a public health emergency of international concern (on 30 January 2020), multiple researchers emphasised the central importance of addressing the effects of the rapidly developing pandemic on population mental health [[1], [2], [3], [4]]. Public health measures adopted in many countries to restrict the spread of SARS-CoV-2 (the virus causing COVID-19), including mandatory quarantine, lockdowns, physical distancing, and closure of non-essential services, resulted in severe social and economic disruption, increasing exposure to several established risk factors for poor mental health (e.g., unemployment, financial hardship, social isolation, loneliness). Additionally, SARS-CoV-2 infection was reported to produce a range of acute and post-acute neuropsychiatric symptoms, including fatigue, sleep disturbance, cognitive impairment, anxiety, and depression, particularly in patients with more severe disease [5]. At the Brain and Mind Centre, in early 2020, we developed a dynamic model for Australia that could be used to simulate the effects of COVID-19-related unemployment, underemployment, and social dislocation on a range of population mental health outcomes, as well as the potential impacts of alternative policy interventions aimed at mitigating these effects. Initial modelling (released in July 2020) [6] indicated that over the period 2020–2025, the COVID-19 pandemic would result in an 11.4% increase in the total number of mental health-related emergency department (ED) presentations, a 12.3% increase in the total number of intentional self-harm hospitalisations, and a 13.7% increase in suicide mortality (i.e., nationally). Nevertheless, the probable magnitude and duration of the economic and psychological effects of the pandemic were, at the time, virtually unknown, so these projections were necessarily dependent upon highly uncertain estimates for several critical model parameters.

As the COVID-19 pandemic has progressed and empirical data on its social, economic, and health impacts have accumulated, it has become clear that our initial model projections overestimated the effects of infection control measures (lockdowns, physical distancing, school closures, etc.) on population mental health. Although a recent, comprehensive meta-analysis of data from 48 studies indicated that COVID-19 has had a substantial impact on mental health globally, increasing the prevalence of major depressive and anxiety disorders by *c*. 25−30% in 2020, national estimates of the effect of the pandemic on disorder prevalence varied considerably, and were significantly lower for countries reporting relatively low daily SARS-CoV-2 infection rates, including Australia [7]. Moreover, despite the substantially increased global burden of common mental disorders, an interrupted time series analysis of suicide mortality in 33 countries provided no evidence for a general increase in suicide rates over the period since the pandemic was declared, although again, significant variation in the effect of COVID-19 was observed across regions [8]. While the overall mental health impact of COVID-19-related social and economic disruption in Australia appears have been relatively modest [9,10], it should be emphasised that the results of several recent analyses focussing on children and adolescents provide greater cause for concern [[11], [12], [13]]. Among 13−17-year-old girls in New South Wales, for example, growth in the ED presentation rate for intentional self-harm or suicidal ideation was reported to increase abruptly during the first two years of the pandemic, from 7.7% per year over the period 2015–2019 to 47.1% per year in 2020 and 2021 [13].

Recently, Glozier et al. proposed four possible explanations for the failure of our modelling to accurately predict the population mental health impacts of COVID-19 in Australia, focussing specifically on our projections for suicide mortality: 1) government intervention, particularly the introduction of national programs aimed at restricting job loss and reducing financial hardship (the JobKeeper Payment and Coronavirus Supplement), minimised the economic impacts of public health measures on individuals; 2) the psychological effects of COVID-19-related social disruption were less severe than expected; 3) our assumption that unemployment, psychological distress, and suicide are causally related does not hold in reality; and 4) suicidal behaviour is simply too complex to model reliably [14]. Here, we critically review the analyses presented in our initial report [6], examining these and other potential explanations for the divergence of our model predictions from observed outcomes, as well as the implications of this divergence for our conclusions about the effectiveness of alternative policy responses (the principal focus of the report), and for the prospect of developing improved models capable of accurately forecasting the mental health impacts of inevitable future threats to public health (including economic crises, natural disasters, global pandemics, etc.) as they arise.

## 2 Methods

### 2.1 Baseline (pre-COVID-19) model

The dynamic model we developed to examine the effects of COVID-19 on population mental health comprises a core set of interconnected sub-models, or sectors, that includes: 1) a population sector, capturing changes in population size and structure resulting from births, migration, ageing, and mortality; 2) a psychological distress sector that models flows of people to and from a state of moderate to very high psychological distress (corresponding to a Kessler 10 score of 16–50); 3) a developmental vulnerability sector, modelling exposure to childhood adversity and its effect on the risk of developing moderate to very high psychological distress in adolescence and adulthood; 4) an education and training sector that captures post-secondary education and vocational training enrolment and completion rates; 5) an employment sector, capturing changes in labour force status in the working-age population (15−64-year-olds); 6) a health services sector, modelling the movement of patients through a network of possible service pathways involving (potentially) general practitioners, community-based mental health services (including psychiatrists, clinical psychologists and other allied health providers, and hospital outpatient services), emergency departments, general and psychiatric hospital inpatient care, and online (self-help) services; and 7) a suicidal behaviour sector that captures hospitalisations for intentional self-harm and suicide mortality (see Fig. S1). Detailed descriptions of all model sectors are provided in Supplementary appendix 1.

Parameter values that could not be derived directly from available data or published research were estimated via constrained optimisation, using historical time series data for a wide range of sociodemographic and health-related outcomes, including unemployment and underemployment rates, the prevalence of moderate to very high psychological distress, intentional self-harm hospitalisation and suicide mortality rates, and rates of mental health services usage (numbers of mental health-related ED presentations, community-based mental health services consultations, psychiatric and general hospital admissions, etc. per year) (see Supplementary appendix 1). Data for the period 1 January 2011 to 31 December 2019 (immediately prior to the first confirmed Australian COVID-19 case being identified on 25 January 2020) were used for the optimisation analysis. Powell's method [15] was employed to obtain the set of (optimal) parameter values minimising the mean of the absolute differences between the observed time series values and the corresponding model outputs, where each difference was expressed as a percentage of the observed value (i.e., the mean absolute percent error was used as the objective function; see ref. [16]). As a means of assessing the ability of the model to predict (as opposed to simply fit) pre-COVID-19 rates of suicidal behaviour, we also estimated all unknown parameter values using data for the period 1 January 2011 to the beginning of 2015 (the training data), and then compared simulated numbers of intentional self-harm hospitalisations and suicides per year derived from the fitted model with empirical estimates for the period 2016 to 2020 (the test data).

### 2.2 Modelling COVID-19 impacts

The demographic, economic, health services, and psychological effects of the continuing COVID-19 pandemic were modelled as abrupt changes in multiple flows directly affected by infection control measures (lockdowns, physical distancing, international travel restrictions, etc.), including: 1) a decrease in the number of people arriving from overseas per year; 2) increases in the per capita rates at which people transition from employment (including underemployment) to unemployment and from full employment to underemployment; 3) reductions in per capita rates of non-acute mental health services provision (including general practitioner services, psychiatrist and allied health services, public hospital outpatient services, and private mental health services); and 4) an increase in the incidence of moderate to very high psychological distress resulting from social dislocation unrelated to job loss (e.g., working from home, not participating in recreational activities, restricted social gatherings) and anxiety about potential unemployment (see ref. [17]). Parameter values determining the scale and duration of these direct effects, which were assumed to begin on 1 March 2020 and decay over time, were estimated via constrained optimisation (as described for the baseline model in the previous section), using population projections for 2025 and 2030 reported by Charles-Edwards et al. [18] and data on labour force status, psychological distress, and government (Medicare)-subsidised mental health services usage published by the Australian Bureau of Statistics [19,20] and the Australian Institute of Health and Welfare [21].

National survey data for the period after mid-March 2020, when stringent infection control measures were first introduced in Australia, indicate that after increasing significantly in the initial months of the pandemic, the prevalence of psychological distress fell relatively rapidly as public health restrictions eased and unemployment and underemployment declined towards pre-pandemic levels in late 2020 and early 2021 [20,22,23]. This suggests that a subset of people experiencing increased psychological distress due to COVID-19-related social and economic disruption did not develop persistent symptoms and had the capacity to recover quickly when the direct effects of the pandemic subsided. As a means of accommodating this distressed but resilient subpopulation, the per capita spontaneous recovery rate (the rate at which people with a Kessler 10 score of 16 or more transition to lower levels of psychological distress independently of receiving care) was assumed to increase as the prevalence of moderate to very high psychological distress increased above that observed immediately prior to 1 March 2020 (see Supplementary appendix 2). Additionally, we assumed that the risk of intentional self-harm and suicide among people developing moderate to very high psychological distress due to the pandemic increased gradually (rather than immediately; see Supplementary appendix 2), consistent with evidence for an association between unemployment duration and suicidal behaviour, and for a delay in the impact of economic recessions on suicide mortality [24,25].

### 2.3 Model analysis

**Fig. 1:**
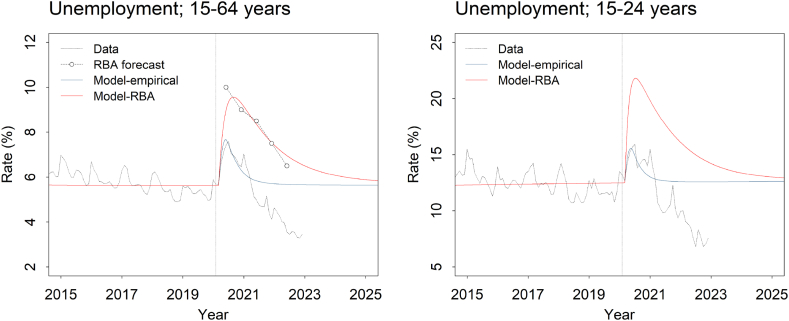
Simulated unemployment rates for Model-empirical and Model-RBA (see Table 1 for details). Monthly Labour Force Survey data (Data) published by the Australian Bureau of Statistics [19] were used to fit Model-empirical; Model-RBA was fitted to the Reserve Bank of Australia's May 2020 unemployment forecast (RBA forecast) [26]. Simulated unemployment rates for Model-ABS are nearly identical to those for Model-empirical. The dotted vertical line in each plot indicates the start of the COVID-19 pandemic (30 January 2020).

Potential explanations for the divergence of our initial model predictions from actual population mental health outcomes were assessed by comparing simulation results for a set of progressively more refined models (see Table 1) with empirical data on the prevalence of moderate to very high psychological distress and numbers of suicides, intentional self-harm hospitalisations, and mental health-related ED presentations published after our modelling was released in July 2020 [6]. Model-RBA in Table 1 corresponds to the model used for the analyses presented in our initial report. Parameter values determining the effect of the pandemic on the national unemployment rate were derived using forecasts published by the Reserve Bank of Australia (RBA) in May 2020 (see Fig. 1) [26], and the incidence of moderate to very high psychological distress was assumed to increase as a direct result of a 10% decrease in social connectedness (note that in early 2020, we had no data to support this predicted decrease in social connectedness; it represents our best estimate at the time). Significantly, for this model we assumed a constant per capita spontaneous recovery rate and an immediate effect of increased psychological distress on the intentional self-harm hospitalisation rate and suicide mortality; i.e., people becoming psychologically distressed due to COVID-19-related social and economic disruption were assumed to recover spontaneously at the same rate as people experiencing moderate to very high psychological distress prior to the pandemic, and to have the same risk of suicidal behaviour.

**Table 1:** Models compared in the analyses.

1. Model-RBA
Unemployment — Model fitted to national unemployment rate projections published by the Reserve Bank of Australia in its May 2020 Statement on Monetary Policy [26].
Psychological distress — Incidence of moderate to very high psychological distress assumed to increase as a direct result of a 10% decrease in social connectedness; per capita spontaneous recovery rate assumed to be identical for everyone experiencing moderate to very high psychological distress.
Suicidal behaviour — Per capita intentional self-harm rate assumed to increase immediately for people developing moderate to very high psychological distress due to COVID-19.
Policy interventions — None.
Notes — Corresponds to the model used for the analyses presented in ref. [6]. No effect of COVID-19 on migration or access to mental health services.
2. Model-ABS
Unemployment — Model fitted to monthly data on age-specific unemployment and underemployment rates over the period March 2020 to December 2022 [19].
Psychological distress — Incidence of moderate to very high psychological distress assumed to increase as a direct result of a 10% decrease in social connectedness; per capita spontaneous recovery rate assumed to be identical for everyone experiencing moderate to very high psychological distress.
Suicidal behaviour — Per capita intentional self-harm rate assumed to increase immediately for people developing moderate to very high psychological distress due to COVID-19.
Policy interventions — Employment and income support programs (JobKeeper Payment and Coronavirus Supplement); Better Access Pandemic Support measure.
Notes — COVID-19 assumed to reduce overseas arrivals and access to non-acute mental health services.
3. Model-empirical
Unemployment — Model fitted to monthly data on age-specific unemployment and underemployment rates over the period March 2020 to December 2022 [19].
Psychological distress — Model fitted to estimates of the prevalence of moderate to very high psychological distress from the Household Impacts of COVID-19 Survey [20]; per capita spontaneous recovery rate assumed to differ for people developing moderate to very high psychological distress due to COVID-19.
Suicidal behaviour — Per capita intentional self-harm rate assumed to increase gradually for people developing moderate to very high psychological distress due to COVID-19.
Policy interventions — Employment and income support programs (JobKeeper Payment and Coronavirus Supplement); Better Access Pandemic Support measure.
Notes — Structurally identical to the model described in ref. [42]. COVID-19 assumed to reduce overseas arrivals and access to non-acute mental health services.

**Fig. 2:**
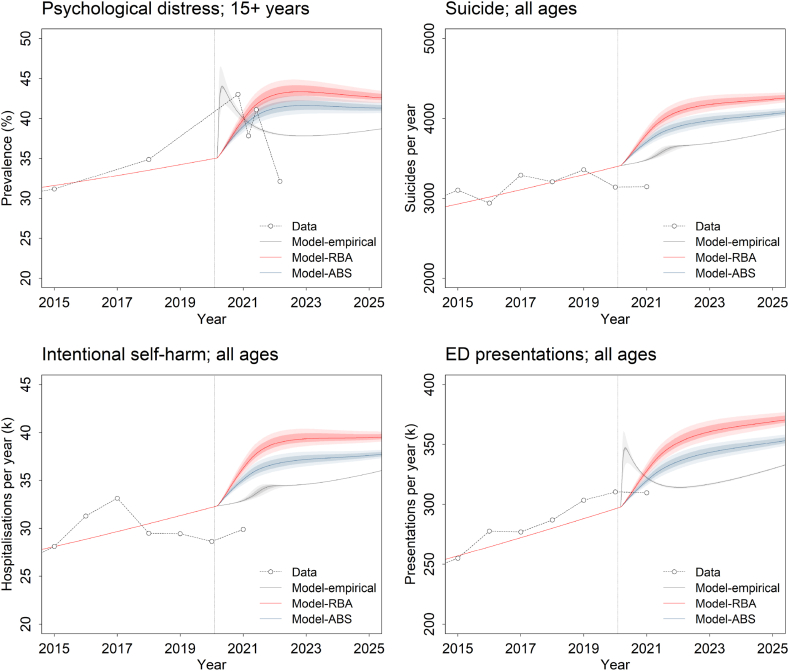
Simulated prevalence of moderate to very high psychological distress (15 years and above) and numbers of suicides, intentional self-harm hospitalisations, and mental health-related emergency department (ED) presentations per year for the three models described in Table 1. Pointwise 50% and 95% intervals derived from the sensitivity analyses (see Methods) are indicated with dark and light shading, respectively. Data on the prevalence of moderate to very high psychological distress are from the National Health Survey (pre-pandemic) [27] and the Household Impacts of COVID-19 Survey [20]; data for the remaining outcomes were obtained from online reports published by the Australian Institute of Health and Welfare [28,43]. The vertical dotted line in each plot indicates the start of the COVID-19 pandemic (30 January 2020). Results for adolescents and young adults (15−24-year-olds) are in Supplementary appendix 4.

Model-10.13039/501100022921ABS is identical to Model-RBA, except that: 1) parameter values determining the effects of COVID-19 on employment were derived (via constrained optimisation; see above) from monthly data on age-specific unemployment and underemployment rates over the period March 2020 to December 2022 (see Fig. 1) [19]; and 2) we explicitly modelled the effects of government interventions introduced to mitigate the economic and mental health impacts of the pandemic (including the JobKeeper Payment and Coronavirus Supplement, and the Better Access Pandemic Support measure), as well as the effects of restricted international travel and reduced overall access to community-based mental health services (see Table 1 and Supplementary appendix 2). The final model, Model-empirical, is the same as Model-ABS, except that: 1) the per capita spontaneous recovery rate was assumed to increase as the prevalence of moderate to very high psychological distress increased above the level observed immediately prior to 1 March 2020; 2) the values of parameters determining the effects of COVID-19-related social disconnection on psychological distress (including the assumed increase in the per capita spontaneous recovery rate) were estimated using national data on the prevalence of moderate to very high psychological distress for the period November 2020 to March 2022 (see Fig. 2) [20]; and 3) the risk of suicidal behaviour among people developing moderate to very high psychological distress due to the pandemic was assumed to increase gradually over time (see previous section and Table 1).

Assuming that in the absence of a job support program (the JobKeeper Payment), national unemployment would have increased to the level predicted by the RBA in May 2020, differences between simulation outputs for Model-RBA and Model-10.13039/501100022921ABS can be attributed predominantly to the effectiveness of government interventions in moderating the impacts of public health measures on employment, and therefore provide a measure of the extent to which early policy responses could have led to the divergence of our model projections from observed outcomes. Differences between simulated outcomes derived from Model-ABS and Model-empirical primarily reflect the combined effects of any discrepancy between the predicted and observed impacts of social disruption on psychological distress and the inclusion of a delay in the effect of increasing psychological distress on rates of intentional self-harm and suicide.

### 2.4 Policy testing and sensitivity analyses

The implications of discordance between our initial modelling results and the observed mental health impacts of COVID-19 for our conclusions about the potential effectiveness of alternative policy responses were assessed by comparing percentage reductions in projected numbers of suicides, intentional self-harm hospitalisations, and mental health-related ED presentations for selected health promotion and services interventions calculated using each model in Table 1. Detailed descriptions of the interventions considered (those projected to have non-negligible effects in our initial modelling) are provided in Supplementary appendix 3. Sensitivity analyses were performed to examine the impact on simulation outputs of uncertainty in estimates of the direct effects of each intervention on access to services and treatment effectiveness, the direct effects of COVID-19-related increases in social disconnection and unemployment on the incidence of psychological distress, and the delay in the effect of increasing psychological distress on rates of intentional self-harm and suicide. Latin hypercube sampling was employed to generate 100 sets of values for all relevant parameters from a uniform joint distribution spanning±20% of the default values (for the delay in the effect of increasing distress on suicidal behaviour, we generated a series of graphical functions specifying the relationship between time since 1 March 2020 and the relative risk of intentional self-harm, as described in Supplementary appendix 2). Modifying the effects of social disconnection and unemployment on the incidence of moderate to very high psychological distress alters the fit of each model to observed (or, for Model-RBA, forecasted) employment and mental health outcomes, so for each set of parameter values, we recalibrated each model before simulating a reference scenario (i.e., no interventions) and all intervention scenarios. Percentage reductions in total numbers of suicides, intentional self-harm hospitalisations, and mental health-related ED presentations for the period 1 January 2020 to the beginning of 2025 were calculated for each set of parameter values and summarised using simple descriptive statistics.

## 3 Results

**Fig. 3:**
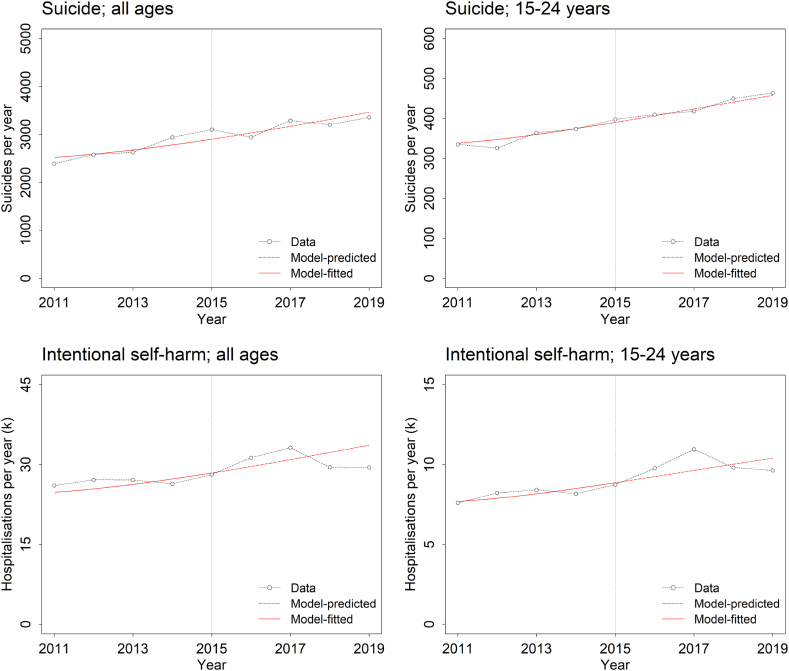
Model-based and empirical estimates of suicide and intentional self-harm hospitalisation rates (all ages and 15−24-year-olds) for the pre-COVID-19 period. Model-based estimates were obtained by fitting the baseline model to data for the period 2011–2015 and predicting suicide and intentional self-harm hospitalisation rates for 2016–2019. Empirical estimates (Data) were derived from the Australian Institute of Health and Welfare's Suicide and Self-harm Monitoring web report [28]. The vertical dotted line in each plot indicates the start of the prediction period (i.e., 2015).

Predicted suicide and intentional self-harm hospitalisation rates per year for the period 2016 to 2019 generated using the baseline (pre-COVID-19) model (fitted to data for 2011 to 2015 only) are generally within *c*. 5% of empirical estimates, although discrepancies between the model predictions and observed values for some years are more substantial (up to 14.2% of the empirical estimate for the total number of intentional self-harm hospitalisations in 2019; Fig. 3). Fig. 2 presents simulation results for the period after the COVID-19 pandemic was declared in early 2020, obtained using each of the models in Table 1 (here, the baseline model was calibrated using data for 2011 to 2019). Model-RBA predicts an increase in the prevalence of moderate to very high psychological distress from 35.0% at the beginning of March 2020 to 43.3% in late 2022, followed by a gradual decline to 42.7% at the start of 2025. Data from the Household Impacts of COVID-19 Survey [20], by comparison, indicate that the prevalence of moderate to very high psychological distress reached a peak in 2020 and then decreased much more rapidly than predicted by Model-RBA, declining from 43.0% in November 2020 to 32.1% in March 2022 (less than the latest directly comparable pre-COVID-19 estimate of 34.9% for 2017–18; see ref. [27]). Projected numbers of suicide deaths, intentional self-harm hospitalisations, and mental health-related ED presentations per year increase continuously, although at a decreasing rate, over the period 1 March 2020 to the start of 2025, and are substantially higher than empirical estimates available for 2021 [21,28].

Model-ABS yields simulated trajectories for moderate to very high psychological distress, suicides, intentional self-harm hospitalisations, and mental health-related ED presentations qualitatively similar to those for Model-RBA, although the projected values at each time point (i.e., after 1 March 2020) are consistently lower (see Fig. 2). In contrast, Model-empirical predicts a substantially more rapid increase and decline in the prevalence of moderate to very high psychological distress, consistent with the Household Impacts of COVID-19 Survey data (which were used to fit the model), a similarly rapid increase and decline in the mental health-related ED presentation rate, and only a relatively slight, transitory increase in suicide and intentional self-harm hospitalisation rates (see Fig. 2). Projections derived from Model-RBA indicate that over the period 1 March 2020 to the beginning of 2025, COVID-19 will result in a 10.3% increase (95% interval 8.4%–12.8%) in the total (cumulative) number of suicides, a 10.6% increase (8.8%–13.1%) in the total number of intentional self-harm hospitalisations, and a 9.7% increase (8.0%–12.0%) in the total number of mental health-related ED presentations (differences between these values and the results presented in our initial report are due primarily to recalibration of the baseline model using pre-COVID-19 data published after the report was released). Corresponding increases for Model-ABS are 6.1% (4.4%–8.0%) for suicides, 5.7% (4.0%–7.6%) for intentional self-harm hospitalisations, and 5.4% (3.9%–7.3%) for mental health-related ED presentations. Model-empirical projects a 0.2% decrease (−0.1%−0.5%) in total suicide mortality, attributable to reduced population growth (a result of COVID-19-related restrictions affecting overseas travel), a 0.3% decrease (0.1%–0.7%) in the total number of intentional self-harm hospitalisations, and a 1.2% increase (1.0%–1.3%) in the total number of mental health-related ED presentations.

**Fig. 4:**
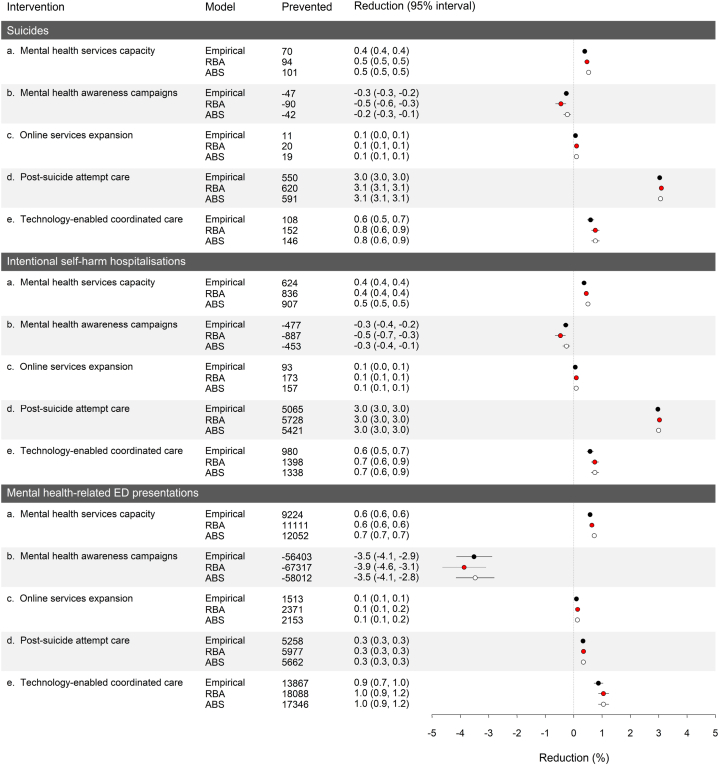
Reductions in total (cumulative) numbers of suicides, intentional self-harm hospitalisations, and mental health-related emergency department (ED) presentations for selected mental health promotion and services interventions calculated using each of the models in Table 1. Descriptions of each intervention are provided in Supplementary appendix 3. Mean numbers of suicides, hospitalisations, and ED presentations prevented (reported in the third column) and mean percentage reductions and 95% intervals (fourth column and plot) were derived from the distributions of outcomes calculated in the sensitivity analyses (see Methods section for details). Note that the 95% intervals provide a measure of the impact of uncertainty in the assumed intervention effects but should not be interpreted as confidence intervals.

Fig. 4 presents percentage reductions in total numbers of suicides, intentional self-harm hospitalisations, and mental health-related ED presentations for selected health promotion and services interventions calculated using each of the models in Table 1. Projected reductions for Model-RBA differ from those presented in our initial report due to recalibration of the baseline model using additional pre-COVID-19 data published after July 2020 and a difference in the intervals over which the intervention and reference scenarios were compared (1 January 2020 to 1 January 2025 in the current analyses, 1 March 2020 to 1 March 2025 in the report; see ref. [6]); however, these differences do not substantively alter our conclusions about the effectiveness of the specific interventions considered here. Post-suicide attempt care is significantly more effective in preventing suicidal behaviour than the remaining interventions, reducing total numbers of suicides and intentional self-harm hospitalisations by 3.09% (3.08%–3.10%) and 3.03% (3.02%–3.03%), respectively. Technology-enabled coordinated care and increases in community-based mental health services capacity reduce total suicide mortality by 0.5–0.9%, total numbers of intentional self-harm hospitalisations by 0.4–0.9%, and total numbers of mental health-related ED presentations by 0.6–1.2%, while the effects of increasing access to online services are more limited (reductions of *c*. 0.1% are projected for all outcomes). Projected increases in total numbers of suicides, intentional self-harm hospitalisations, and mental health-related ED presentations observed for public awareness campaigns are attributable (at least partially) to increased disengagement and associated adverse effects on mental health resulting from the inability of capacity-constrained services to accommodate significant increases in help seeking behaviour [6]. Despite the substantial differences in the reference simulations for the three models (Fig. 2), Model-ABS and Model-empirical yield projected intervention effects very similar to those for Model-RBA.

## 4 Discussion

The simulation results presented in Fig. 2 indicate that our overestimation of the impacts of COVID-19-related social and economic disruption on population mental health is due predominantly to our assumption (made implicitly) that new cases of moderate to very high psychological distress emerging as a result of the pandemic could be considered equivalent to pre-pandemic cases (i.e., with identical per capita rates of spontaneous recovery, intentional self-harm, etc.). Allowing per capita rates of spontaneous recovery and suicidal behaviour to differ for people developing moderate to very high psychological distress as a result of COVID-19 substantially improves the fit of our model to empirical estimates of the prevalence of psychological distress for the period after the pandemic was declared, and leads to markedly lower predicted effects of the pandemic on total numbers of suicides, intentional self-harm hospitalisations, and mental health-related ED presentations (see results for Model-empirical; Fig. 2). Notable differences between the simulations for Model-RBA and Model-10.13039/501100022921ABS (which are fitted to projected and empirical unemployment rates, respectively) suggest that government intervention aimed at moderating the impacts of infection control measures on employment also contributed to the divergence of our projections from actual outcomes, although the magnitude of this contribution is relatively small (note that our initial modelling indicated that employment support programs would reduce suicide mortality by 2.9% over the period 2020–2025, which is less than the reduction of 3.8% implied by the difference between the results for Model-RBA and Model-10.13039/501100022921ABS, but not drastically less; see ref. [6]). In contrast, our simulation analyses provide no evidence that we seriously overestimated the effect of COVID-19-related social disruption on the risk of developing psychological distress; when compared with data from the Household Impacts of COVID-19 Survey, the simulated prevalence of moderate to very high psychological distress for Model-RBA increases more slowly and reaches a very similar maximum value (see Fig. 2).

Our assumption that the per capita spontaneous recovery rate may differ between people developing moderate to very high psychological distress due to COVID-19-related public health restrictions and those experiencing a comparable level of psychological distress prior to the pandemic (made for Model-empirical only) is consistent with a longitudinal analysis of the natural course of psychological distress in a cohort of 7934 British civil servants followed over a 21-year period [29]. Among the principal findings of this analysis was that a positive dose-response relationship exists between the frequency and/or duration of past self-reported psychological distress (assessed using the 30-item General Health Questionnaire) and the risk of future psychological distress, so that, for example, participants reporting a clinically significant level of psychological distress in only one of the initial seven study phases were substantially less likely to report significant psychological distress in the eighth phase than participants reporting significant psychological distress in three of the initial seven phases. Regardless of the cause of the observed dose-response relationship (increasing sensitivity to frequent, minor stressors or inter-individual variation in relatively stable vulnerabilities attributable to polygenic risk, adverse childhood exposures, etc.), this finding suggests that many people becoming psychologically distressed as a direct result of the pandemic (i.e., those with no history of recurrent or prolonged psychological distress) were at relatively low risk of developing persistent symptoms. As noted above (see Methods), our additional assumption of a gradual increase in per capita rates of intentional self-harm and suicide in this newly distressed subpopulation is consistent with evidence for an association between duration of unemployment and suicidal behaviour, and for a delay in the impact of economic recessions on suicide mortality [24,25].

Glozier et al.’s conclusion that the suicide rate in Australia has remained stable over the past 5−10 years despite an increase in the prevalence of psychological distress, and that this renders our assumption of a causal effect of psychological distress on suicide mortality questionable [14], apparently ignores a clear increasing trend in the national age-standardised suicide rate (per 10^5^ population) between 2005 and 2019 (the latest non-provisional estimate; see ref. [28]), and is inconsistent with substantial evidence from population-based cohort studies indicating that psychological distress increases the risk of suicidal behaviour at the individual level [[30], [31], [32], [33]]. Although evidence from conventional epidemiological studies for a causal association between unemployment and suicide is more equivocal, convergent cross mapping analyses [34] using monthly data on labour underutilisation and suicide rates for Australia provide direct evidence that both unemployment and underemployment were significant drivers of suicide mortality over the period January 2004 to December 2016 [35]. Additional evidence for a causal effect of unemployment on intentional self-harm and suicide comes from meta-analyses of effect estimates from more than 80 longitudinal studies, indicating that transitions from employment to unemployment typically produce an increase in psychological distress, while transitions in the reverse direction are associated with significant improvements in mental health [36]. Accordingly, while the specific causal pathways connecting unemployment, psychological distress, and suicide are only incompletely understood [37], Glozier et al.’s proposal that our overestimation of COVID-19-related suicide mortality may be attributable to a false assumption that psychological distress and unemployment affect suicidal behaviour [14] is not supported by the majority of available evidence.

Although the magnitude of the COVID-19-related increases in adverse mental health outcomes predicted in our initial modelling clearly has implications for the scale and urgency of potential policy responses aimed at restricting the impacts of the pandemic on population mental health, the results presented in Fig. 4 indicate that the divergence of our projections from observed outcomes does not significantly affect our conclusions about the capacity of alternative interventions to reduce numbers of suicides, intentional self-harm hospitalisations, and mental health-related ED presentations. Accordingly, our recommended combination of interventions (namely, employment and education support programs, increasing the capacity of community-based mental health services, technology-enabled care coordination, and post suicide-attempt care) [6] remains unchanged (note that the effects of employment support programs implied by the differences between the simulation results for Model-RBA and Model-10.13039/501100022921ABS are greater than those for the health promotion and services interventions in Fig. 4, consistent with our previous conclusion that minimising unemployment would be the single most effective means of mitigating the adverse mental health impacts of the pandemic; see ref. [6]). This finding is consistent with the results of a more detailed analysis examining the consequences of uncertainty in predictions of the mental health effects of COVID-19 for model-based comparisons of the potential effectiveness of 13 suicide prevention interventions in the Perth South region of Western Australia [38], and provides additional evidence that the utility of dynamic modelling in supporting public health policy and planning decisions is not necessarily dependent upon an ability to accurately predict future population health outcomes.

## 5 Limitations

There are two principal limitations of the analyses presented here that should be noted. First, the available data on population mental health outcomes for the period after strict infection control measures were first introduced in March 2020 are relatively sparse, consisting of estimates of the prevalence of moderate to very high psychological distress for only four time points between November 2020 and March 2022, and yearly numbers of suicides, intentional self-harm hospitalisations, and mental health-related ED presentations for 2020 and 2021 (Fig. 2). Nevertheless, the abrupt increase and rapid decline in the prevalence of moderate to very high psychological distress indicated by the Household Impacts of COVID-19 Survey data used in our analyses (see ref. [20]; Fig. 2) is at least broadly consistent with the results of a recent analysis of daily estimates of the prevalence of anxiety and depressive symptoms in Australia for the period May 2020 to December 2021, which provided evidence for relatively rapid decreases in psychological distress after prolonged lockdowns ended in Victoria and New South Wales [39]. The second limitation relates to our assumption that in the absence of a national job support program (the JobKeeper Payment), the unemployment rate would have increased to the level predicted by the RBA in its May 2020 Statement on Monetary Policy [26]. While this assumption is convenient, the increase in unemployment that would have occurred under the counterfactual scenario (without the JobKeeper Payment) is clearly unknown, so that the population mental health effects of government intervention aimed at preventing job loss may differ considerably from those implied by differences between the simulation results for Model-RBA and Model-10.13039/501100022921ABS.

## 6 Conclusion

The simulation analyses presented here provide evidence that our overestimation of the mental health impacts of COVID-19 in Australia can be attributed predominantly to unmodelled differences in per capita rates of spontaneous recovery and suicidal behaviour between people experiencing moderate to very high psychological distress prior to the pandemic (including those with persistent mental disorders) and people developing comparable levels of psychological distress only as a result of disruptive public health restrictions and other COVID-19-related causes (fear of infection, continual exposure to media reporting [40], etc.). Although a recent review of 64 distinct suicide prediction models demonstrated clearly that suicidal behaviour cannot be reliably predicted at the individual level (at least currently) [41], discrepancies between observed pre-pandemic suicide and intentional self-harm hospitalisation rates and predictions derived from our model are generally small (see Fig. 3), suggesting that Glozier et al.’s conclusion that ‘Suicide modelling is inherently very inaccurate’ [14] does not necessarily apply at the population level. Aside from accommodating the potentially significant influence of prior mental health on the psychological effects of severe social and economic disruption, there are several obvious directions in which our modelling could be extended to better capture the dynamics of population mental health and its underlying causes (representing states of psychological distress and mental disorder in greater detail, explicitly modelling the feedback processes driving aggregate demand and unemployment, incorporating additional social and economic sources of significant psychological stress), so that the prospect of developing substantially improved models capable of supporting effective responses to future public health crises would appear to be high. Certainly, the failure of our preliminary modelling to accurately predict the mental health impacts of COVID-19 does not entail that ‘this area is so complex with so many interactions that [any attempt at] making confident and accurate predictions is … futile’ [14].

## CRediT authorship contribution statement

**Adam Skinner:** Writing – original draft, Formal analysis, Conceptualization. **Jo-An Occhipinti:** Writing – review & editing, Conceptualization. **Yun Ju Christine Song:** Writing – review & editing, Conceptualization. **Ian B. Hickie:** Writing – review & editing, Conceptualization.

## Declaration of competing interest

The authors declare the following financial interests/personal relationships which may be considered as potential competing interests: Associate Professor Jo-An Occhipinti is Head of Systems Modelling, Simulation & Data Science at the Brain and Mind Centre, University of Sydney and Managing Director of Computer Simulation and Advanced Research Technologies (CSART). Professor Ian Hickie (IBH) was an inaugural Commissioner on Australia's National Mental Health Commission (2012–18). He is the Co-Director, Health and Policy at the Brain and Mind Centre, University of Sydney. The Brain and Mind Centre operates an early-intervention youth service at Camperdown under contract to headspace. IBH has previously led community-based and pharmaceutical industry-supported (Wyeth, Eli Lily, Servier, Pfizer, AstraZeneca) projects focused on the identification and better management of anxiety and depression. He was a member of the Medical Advisory Panel for Medibank Private until October 2017, a Board Member of Psychosis Australia Trust, and a member of Veterans Mental Health Clinical Reference group. He is the Chief Scientific Advisor to, and a 3.2% equity shareholder in, InnoWell Pty Ltd. InnoWell was formed by the University of Sydney (45% equity) and PwC (Australia; 45% equity) to deliver the $30 M Australian Government-funded Project Synergy (2017−20; a three-year program for the transformation of mental health services) and to lead transformation of mental health services internationally through the use of innovative technologies. Dr Adam Skinner (AS) and Dr Yun Ju Christine Song (YJCS) declare no competing interests.

## Data Availability

Details of all data sources used for the analyses are provided in the Methods section of the paper and the Supplementary Material.
