# Supplementary material for: Mental health impacts of COVID-19: A retrospective analysis of dynamic modelling projections for Australia
Source: Heliyon. 2024 Mar 16;10(7):e28250. doi: 10.1016/j.heliyon.2024.e28250 (PMC10998102; doi:10.1016/j.heliyon.2024.e28250)
Supplement: Multimedia component 1 [file mmc1.docx]

Supplementary appendices 1−4. Mental health impacts of COVID-19: a retrospective analysis of dynamic modelling projections for Australia

Adam Skinner^1,*^, Jo-An Occhipinti^1,2^, Yun Ju Christine Song^1^, Ian B. Hickie^1^

^1^ Brain and Mind Centre, Faculty of Medicine and Health, University of Sydney, Sydney, Australia; ^2^ Computer Simulation and Advanced Research Technologies (CSART), Sydney, Australia

^*^ Corresponding author. Email: adam.skinner@sydney.edu.au. Address: Level 4, Building M02C, 94 Mallet Street, Camperdown, NSW 2050, Australia. Phone: +61 450 458 201.

Supplementary appendix 1

Baseline model structure

*1.1. Overview*

The system dynamics model used for the simulation experiments presented in the paper is composed of a set of interconnected sub-models, or sectors, that includes: 1) a population sector, capturing changes in population size and structure resulting from births, migration, aging, and mortality; 2) a psychological distress sector that models flows of people to and from a state of moderate to very high psychological distress; 3) a developmental vulnerability sector, modelling exposure to childhood adversity and its effect on the risk of developing mental disorders in adolescence and adulthood; 4) an education and training sector that captures post-secondary education and vocational training enrolment and completion rates; 5) an employment sector, capturing changes in labour force status in the working-age population (15−64-year-olds); 6) a health services sector, modelling the movement of patients through a network of possible service pathways involving (potentially) general practitioners, community-based mental health services (including psychiatrists, clinical psychologists and other allied health providers, and hospital outpatient services), emergency departments, general and psychiatric hospital inpatient care, and online services; and 7) a suicidal behaviour sector that captures intentional self-harm hospitalisations and suicide mortality. A high-level map of the core model showing the (causal) connections among sectors is presented in figure S1.

*1.2. Population sector*

Figure S2 shows the structure of the population sector, which models changes in the size and composition of the Australian population resulting from births, migration, aging, and mortality. The total national population is represented as 5 stocks (i.e., state variables), corresponding to numbers of people aged 0−14 years, 15−24 years, 25−44 years, 45−64 years, and 65 years and above. Population size increases via births (which flow into the stock of 0−14-year-olds) and immigration and decreases through emigration and mortality. Aging is modelled as a first-order delay, in which people flow out of each stock (except the stock of people aged ≥ 65 years) at a rate $n/d$, where $n$ is the number of people in the stock at any particular time point and the delay time $d$ is the mean number of years a person spends in the stock. Births and deaths occur at rates $bP$ and $\theta_{i}mP_{i}$, respectively, where $P$ is the total population, $\theta_{i}$ and $P_{i}$ are, respectively, the mortality hazard ratio and population for age group $i$, and the per capita birth rate $b$ and per capita mortality rate for the total population $m$ decline at constant fractional rates per year. Net migration for age group $i$ is equal to $I_{i}-e_{i}P_{i}$, where $I_{i}$ is age-specific immigration per year and $e_{i}$ is the age-specific per capita emigration rate per year. Population estimates derived from the system dynamics model are presented together with estimates from the Australian Bureau of Statistics (2019) in figure S3.


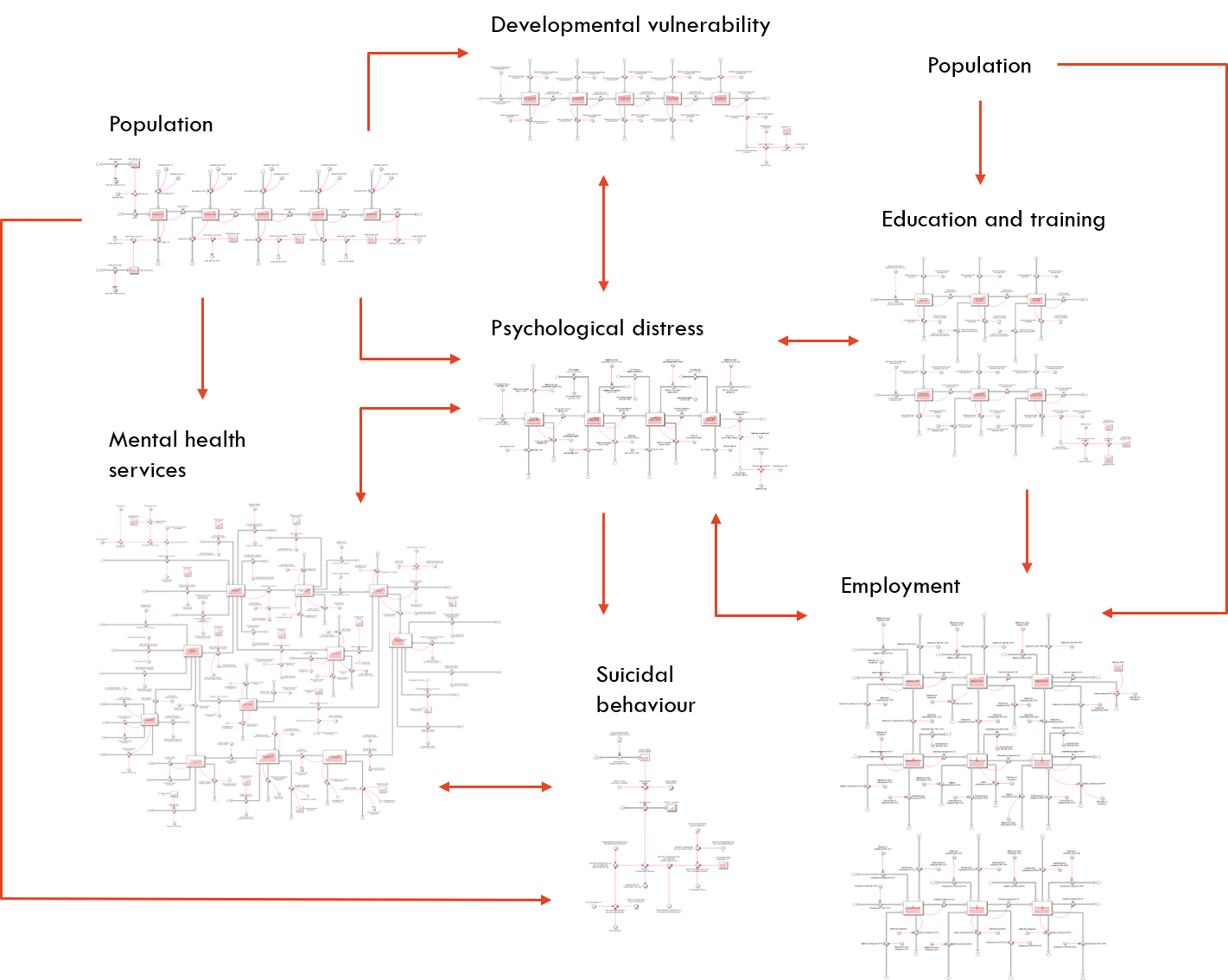


Figure S1. High-level map of the core system dynamics model showing the causal connections among model sectors. Single-headed arrows indicate unidirectional causal connections; bidirectional causal connections are shown as double-headed arrows.

*1.3. Psychological distress sector*

The psychological distress sector captures transitions between states of low psychological distress (Kessler 10 [K10] scores 10−15) and moderate to very high psychological distress (K10 scores ≥ 16) in each age group (figure S4). Numbers of people currently experiencing moderate to very high levels of psychological distress are modelled as stocks with inflows corresponding to psychological distress incidence and outflows corresponding to recovery. Psychological distress incidence is equal to $hsL$, where $s$ is the reference (or base)


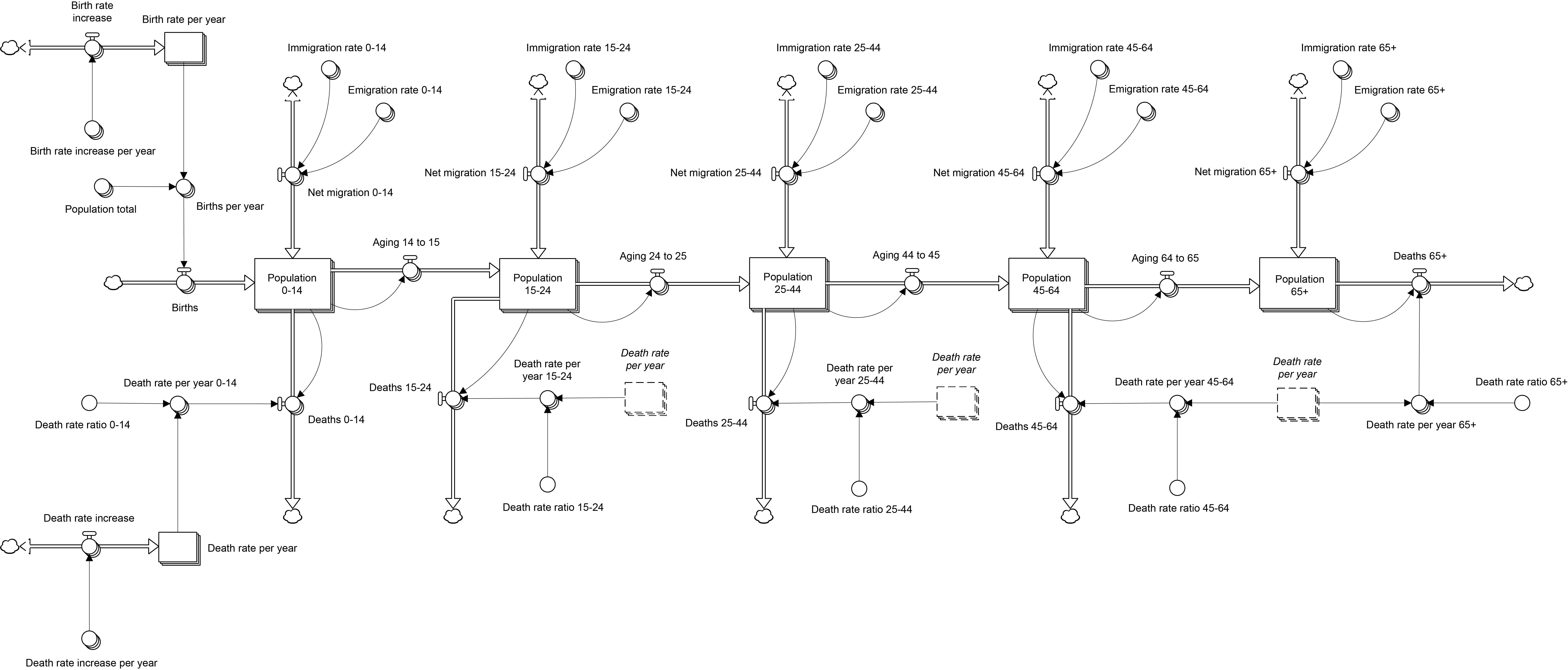


Figure S2. Structure of the population sector.


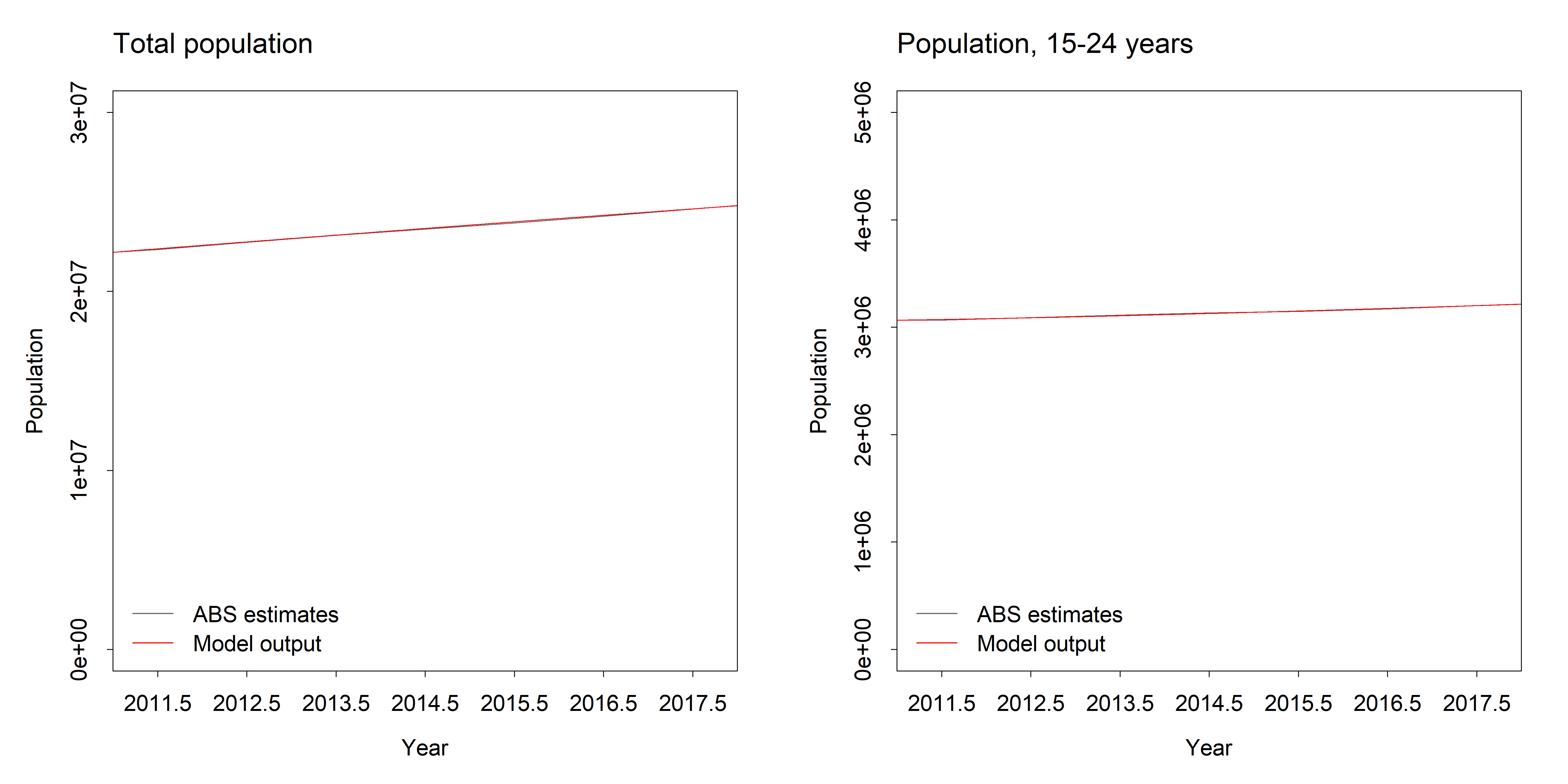


Figure S3. Population estimates (all ages and 15−24-year-olds) derived from the system dynamics model and from the Australian Bureau of Statistics (ABS; 2019).

per capita rate of distress onset per year, $h$ is the product of the effects of developmental vulnerability during childhood (Green et al., 2019), unemployment or (for people aged 15−24 years) non-participation in education or employment (Australian Bureau of Statistics, 2012), and underemployment (Dooley et al., 2000) on psychological distress onset, and $L$ is the number of people experiencing low levels of psychological distress. Moderately to highly distressed people in each age group recover at a yearly rate $rH+T$, where $r$ is the per capita spontaneous recovery rate per year, $H$ is the number of people currently experiencing moderate to very high psychological distress, and $T$ is the number of people moving from a state of moderate to very high psychological distress to a state of low psychological distress per year due to effective mental health treatment (see section 1.7 below). Aging of people experiencing moderate to very high levels of distress is modelled using the same approach described for the population sector above (i.e., as a first-order delay; see section 1.2).

Numbers of people with moderate to very high psychological distress increase (or decrease) via net migration at age-specific rates $p_{i}I_{i}-q_{i}e_{i}P_{i}$, where $p_{i}$ and $q_{i}$ are the age-specific proportions of people with moderate to very high psychological distress among overseas arrivals and Australian residents, respectively, $I_{i}$ is total age-specific immigration per year, $e_{i}$ is the age-specific per capita emigration rate per year, and $P_{i}$ is the number of people in age group $i$ in the Australian population. Age-specific per capita mortality among people with moderate to very high psychological distress is assumed to be 1.37 times that for people with low psychological


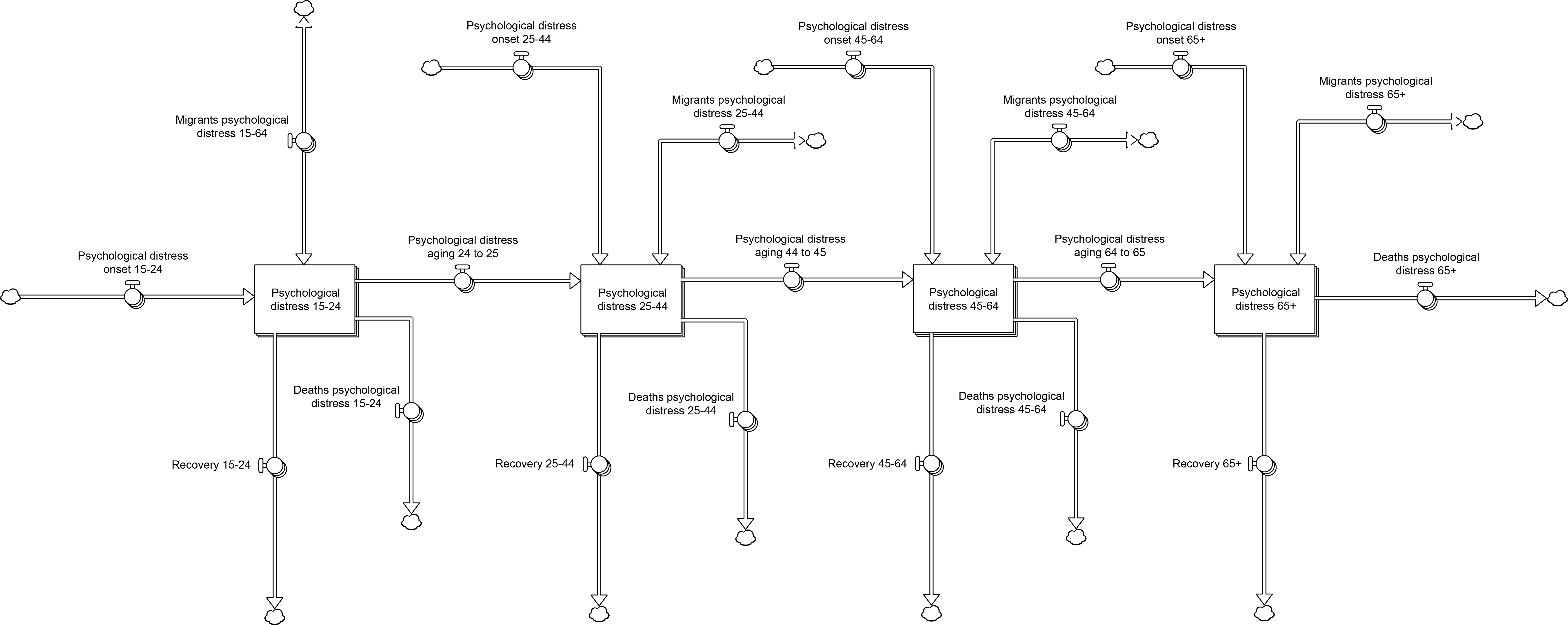


Figure S4. Stock and flow structure of the psychological distress sector.

distress (Russ et al., 2012). Moderate to very high psychological distress prevalence estimates derived from the simulation model and from the National Health Survey (Australian Bureau of Statistics, 2018) are presented in figure S5.


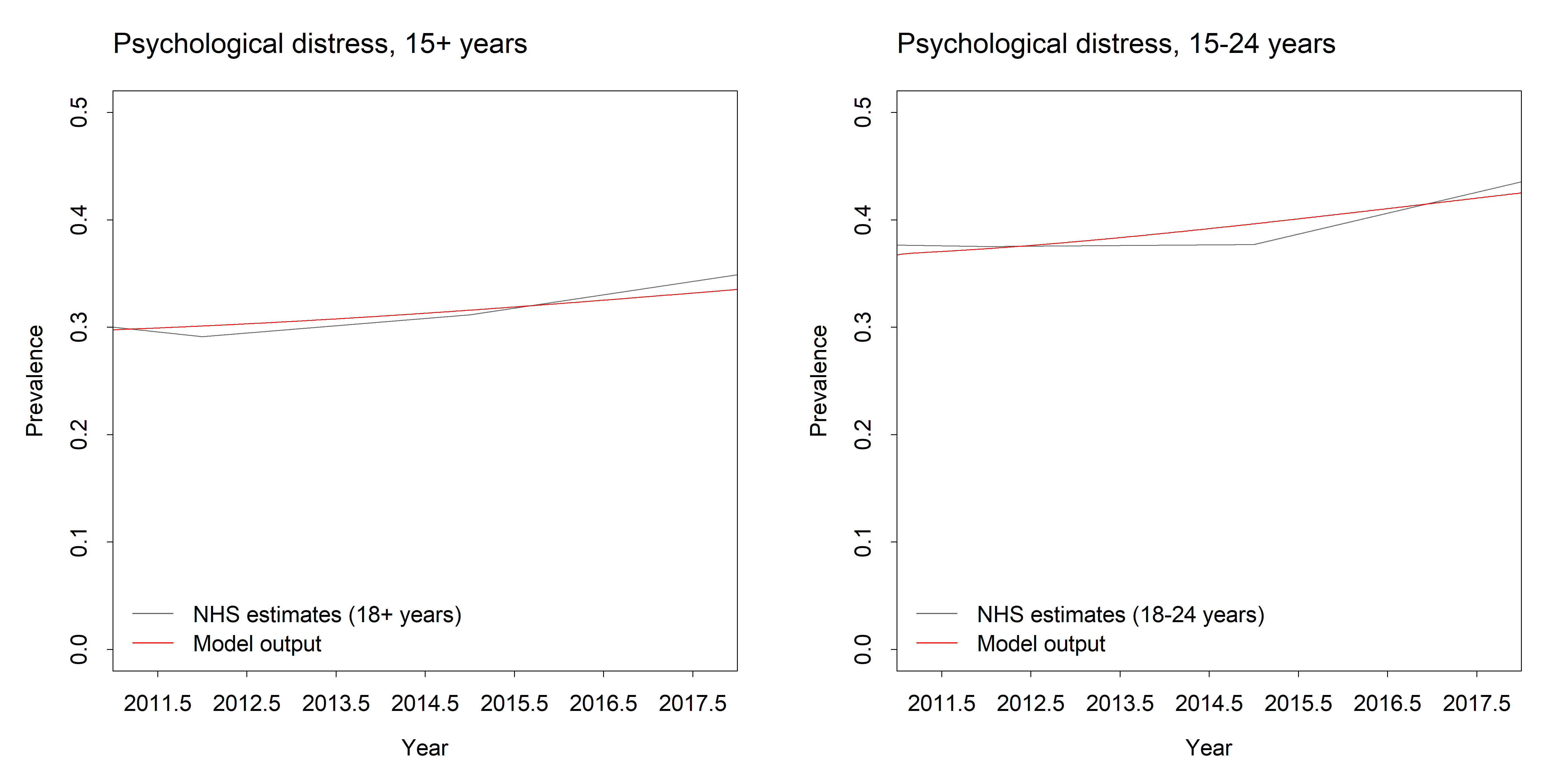


Figure S5. Moderate to very high psychological distress prevalence estimates derived from the dynamic model and from the National Health Survey (NHS; Australian Bureau of Statistics, 2018).

*1.4. Developmental vulnerability sector*

The developmental vulnerability sector models exposure to adversity during childhood and its effect on the risk of developing mental disorders in adolescence and adulthood. The number of developmentally vulnerable children aged 0−14 years is modelled as a stock that increases as children at low risk of psychopathology transition to a state of higher risk (see figure S6); we assume that the onset of psychopathological vulnerability depends on cumulative exposure to adverse experiences (e.g., parental psychological distress, physical and sexual abuse, domestic violence, poverty) and is irreversible. The incidence of significant psychopathological vulnerability is equal to $hsR$, where $s$ is the reference per capita rate at which children at low risk of psychopathology transition to a state of higher risk per year, $h$ is the effect of parental psychological distress on the risk of developing mental disorders in later life (Dean et al., 2018), and $R$ is the number of low-risk 0−14-year-olds in the population. The per capita rate $s$ was set so that the prevalence of psychopathological vulnerability (i.e., for 0−14-year-olds) aligned with estimates of the proportion of developmentally vulnerable children derived from the Australian Early Child Development Census (https://www.aedc.gov.au; figure S7).


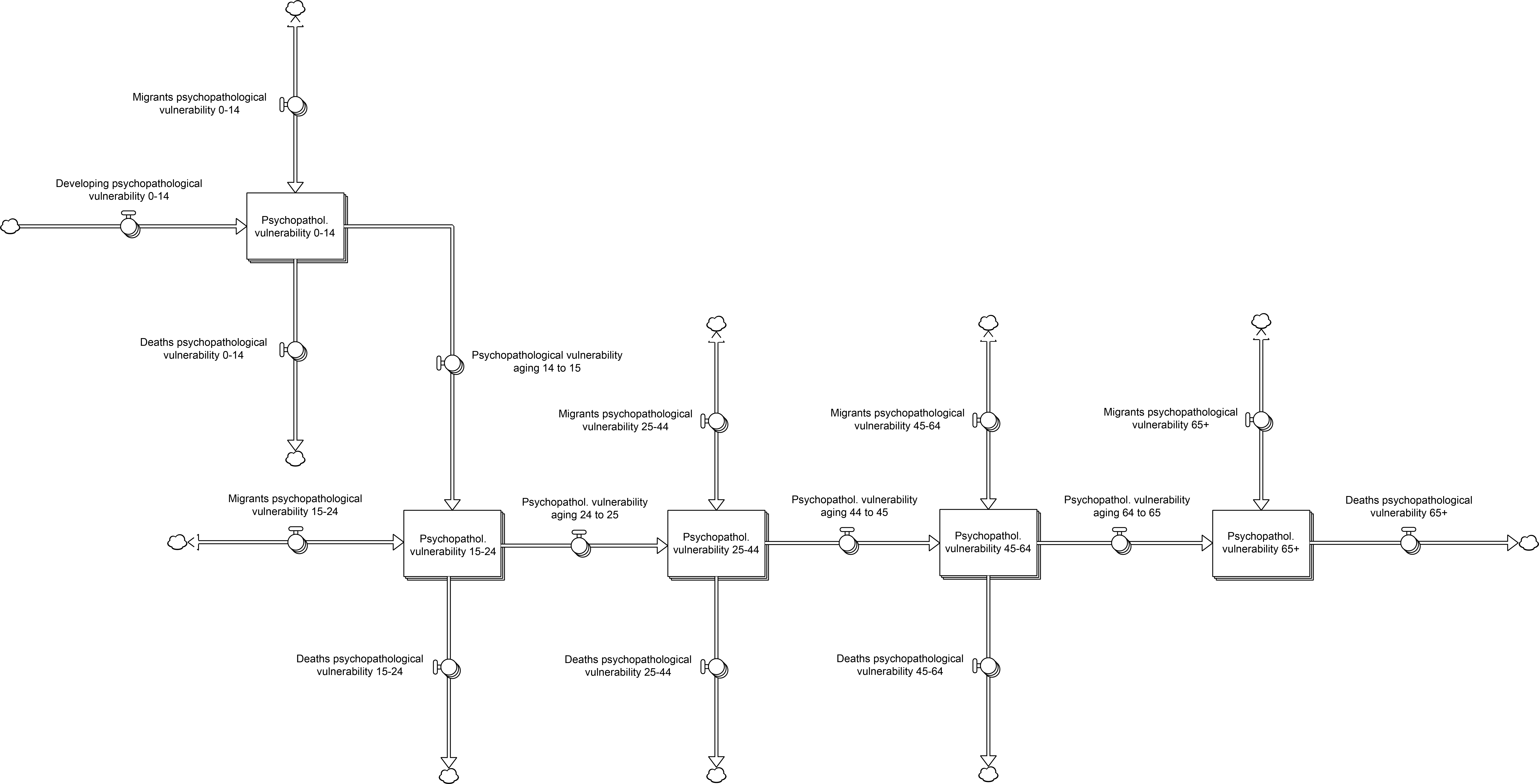


Figure S6. Stock and flow structure of the developmental vulnerability sector.


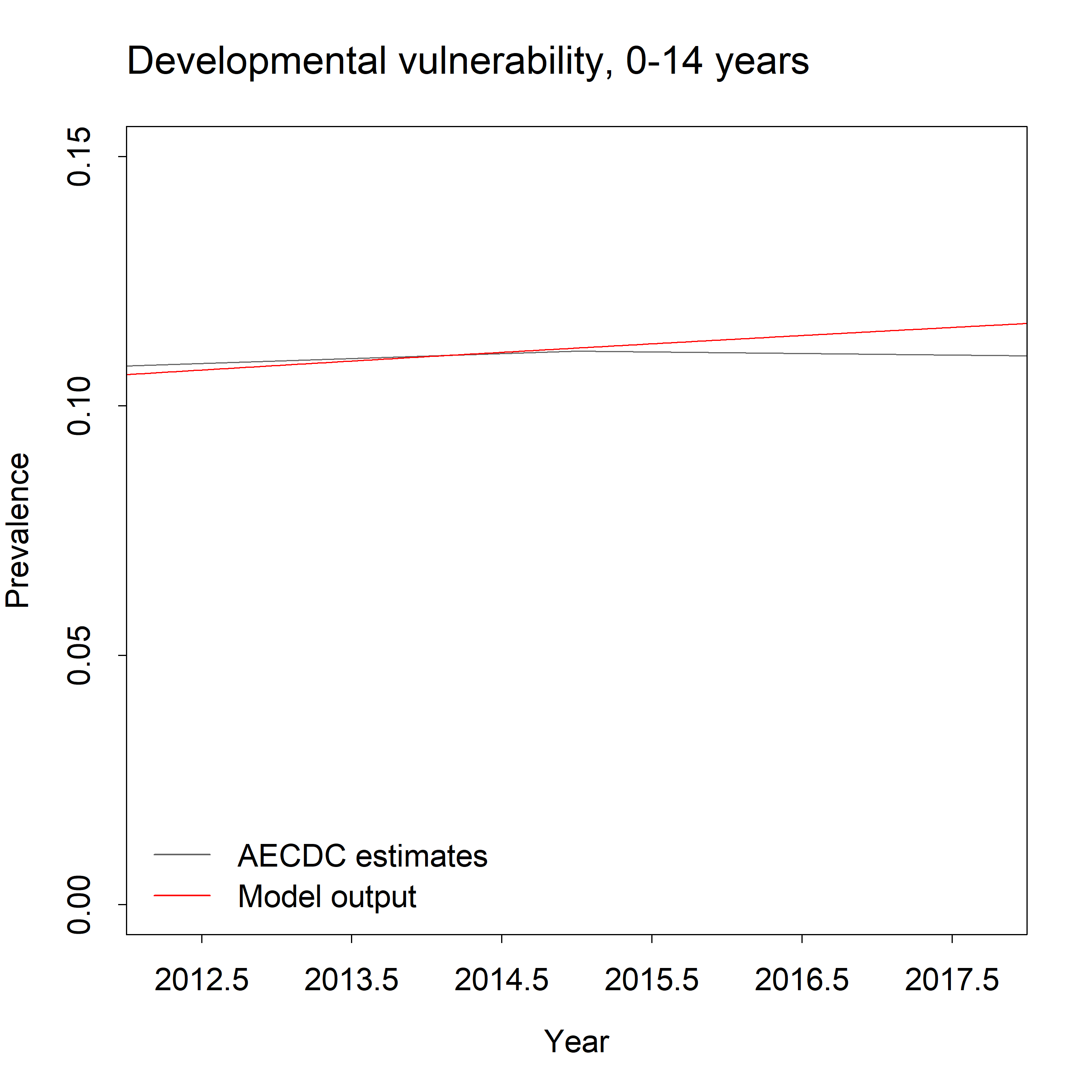


Figure S7. Psychopathological vulnerability prevalence estimates derived from the system dynamics model and from the Australian Early Child Development Census (AECDC; https://www.aedc.gov.au).

As developmentally vulnerable children turn 15, they flow into an aging chain that provides a means of tracking numbers of people aged 15−24 years, 25−44 years, 45−64 years, and 65 years and above at higher risk of developing mental disorders due to adverse exposures in childhood. A person’s level of risk (either high or low) at the time they reach 15 years of age is assumed to remain unchanged (people are removed from the aging chain only through mortality and emigration; see figure S6) and affects the probability that they will experience moderate to very high psychological distress throughout their life (see section 1.3). Age-specific per capita mortality rates for people with a high risk of developing mental disorders are assumed to be 1.37 times those for people with a low level of risk (Russ et al., 2012). Net migration adds to (or subtracts from) the numbers of people at increased risk of moderate to very high psychological distress at age-specific rates $p_{i}I_{i}-q_{i}e_{i}P_{i}$, where $p_{i}$ and $q_{i}$ are the age-specific proportions of people with a higher risk of psychopathology among overseas arrivals and Australian residents, respectively, $I_{i}$ is total age-specific immigration per year, $e_{i}$ is the age-specific per capita emigration rate per year, and $P_{i}$ is the number of people in age group $i$ in the Australian population.

*1.5. Education and training sector*

Figure S8 shows the structure of the education and training sector, which captures post-secondary education and vocational training enrolment and completion rates (certificate III level and above; Australian Bureau of Statistics, 2020) among people aged 15−64 years. Numbers of people currently studying for a post-secondary qualification are modelled as stocks with inflows corresponding to enrolment and outflows corresponding to completion and discontinuation (i.e., dropping out of study prior to completion). Age-specific enrolment rates are calculated as $gkN$, where $k$ is the reference per capita enrolment rate per year, $g$ is the effect of psychological distress on entry into post-secondary study (Lee et al., 2009), and $N$ is the number of people not currently studying. Completion and discontinuation rates are equal to $cS$ and $hdS$, respectively, where $c$ is the per capita completion rate per year, $d$ is the base per capita discontinuation rate per year, $h$ is the effect of psychological distress on the discontinuation rate (Lee et al., 2009), and $S$ is the number of people currently studying for a post-secondary qualification. People undertaking post-secondary study are assumed to experience the same per capita mortality as people not currently studying (i.e., the per capita mortality rates for the total population are applied to the stocks of people enrolled in post-secondary education and vocational training).


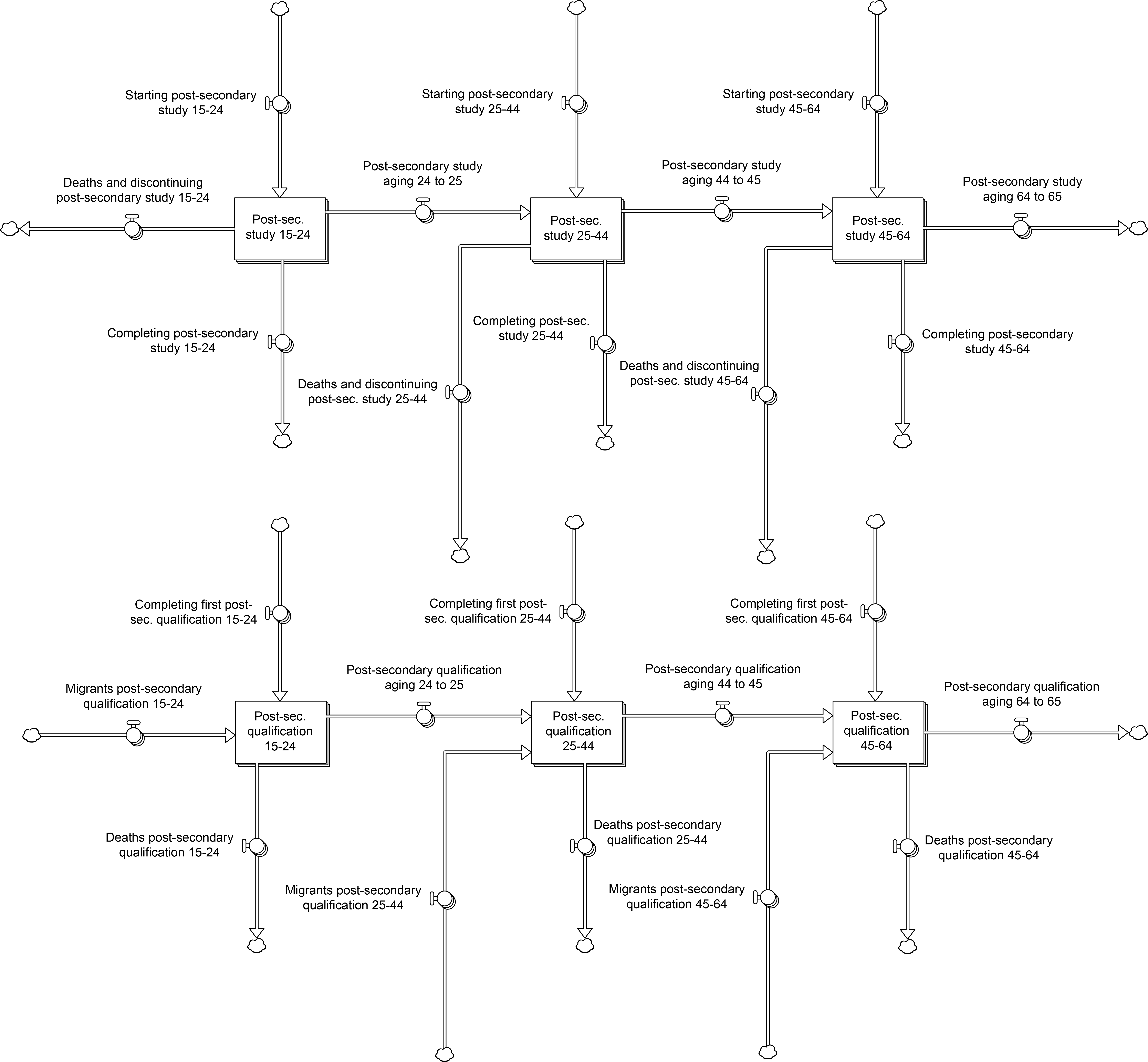


Figure S8. Stock and flow structure of the education sector.

As people complete study, a fraction $f$, corresponding to those without a previous post-secondary qualification, flow into stocks of people aged 15−24 years, 25−44 years, and 45−64 years with at least one qualification. Numbers of people with a post-secondary qualification increase (or decrease) due to net migration at rates equal to $p_{i}I_{i}-q_{i}e_{i}P_{i}$, where $p_{i}$ and $q_{i}$ are the age-specific proportions of people with a post-secondary qualification among overseas arrivals and Australian residents, respectively, $I_{i}$ is total age-specific immigration per year, $e_{i}$ is the age-specific per capita emigration rate per year, and $P_{i}$ is the number of people in age group $i$ in the Australian population. Per capita mortality rates for people with a post-secondary qualification are assumed to be 0.74 times those for people without a qualification (Backlund et al., 1999). Figure S9 presents model-based estimates of the numbers of people enrolled in post-secondary education and vocational training and the proportions of people with a post-secondary qualification, together with estimates derived from the Australian Bureau of Statistics (2020).

*1.6. Employment sector*

The structure of the employment sector, which models labour market transitions in the working-age population (15−64 years), is presented in figure S10. The total labour force is represented as nine stocks, corresponding to the numbers of fully employed, underemployed, and unemployed people aged 15−24 years, 25−44 years, and 45−64 years. Adolescents turning 15 are assumed to enter the population of those not in the labour force (NILF), i.e., people who are neither employed nor seeking employment (15-years-olds are required to attend school full-time in Australia, and the vast majority will not be in the labour force). People not in the labour force may decide to seek employment, at which point they enter the stocks of unemployed people, while those seeking employment (i.e., the unemployed) may leave the labour force. Age-specific net flows from the unemployed population to the population of people not in the labour force are calculated as $fsU-hrN$, where $U$ and $N$ are, respectively, the numbers of unemployed people and people not in the labour force, $s$ is the reference (or base) per capita rate that unemployed people leave the labour force per year, $f$ is the effect of the unemployment rate on labour force participation (assumed to be greater than 1, so that increases in the unemployment rate reduce participation; Mitchell et al., 2019), $r$ is the base per capita rate that people enter the labour force per year, and $h$ is the product of the effects of psychological distress (Frijters et al., 2014) and completion of post-secondary education or vocational training (Australian Bureau of Statistics, 2020) on the labour force entry rate.


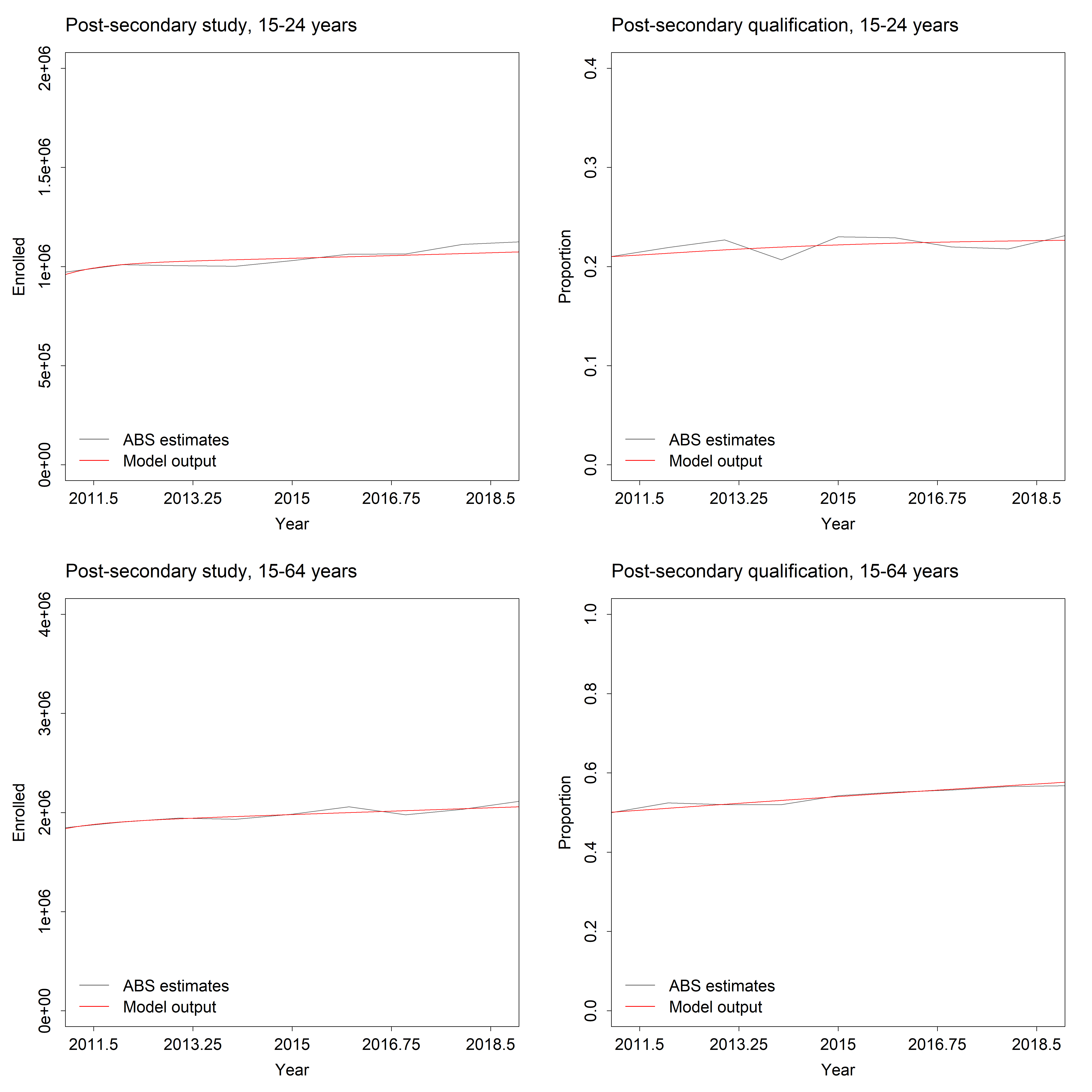


Figure S9. Estimates of numbers of people enrolled in post-secondary education and vocational training and the proportions of people with a post-secondary qualification derived from the system dynamics model and from the Australian Bureau of Statistics (ABS; 2020).

Fully employed people become unemployed at age-specific rates $vE$, where $E$ is the fully employed population and $v$ is the per capita rate of job loss per year, while unemployed people secure full employment at age-specific rates $bwU$, where $U$ is the unemployed population, $w$ is the reference (or base) per capita rate of full employment initiation per year, and $b$ is the product of the effects of psychological distress (Frijters et al., 2014) and completion of post-secondary study (Australian Bureau of Statistics, 2020) on the employment initiation rate; the net yearly flow from unemployment to full employment is therefore $bwU-vE$. Net flows from


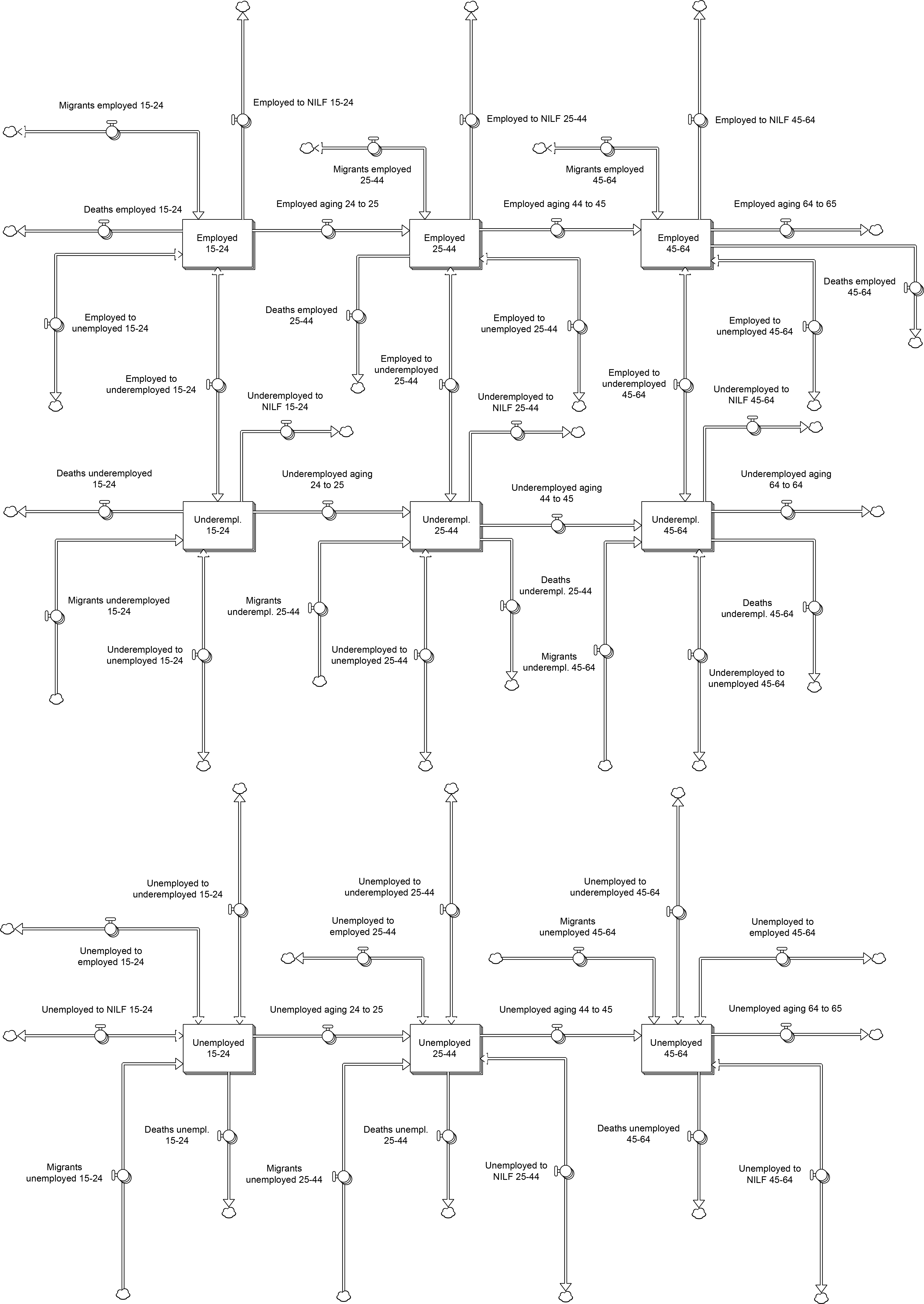


Figure S10. Stock and flow structure of the employment sector.

unemployment to underemployment per year are specified in a similar way (the effects of distress and post-secondary study on transitions to full employment and underemployment are assumed to be the same). Underemployed people (i.e., those in employment working fewer hours per week than they would like) become fully employed at age-specific rates $ckM$, where $M$ is the underemployed population, $k$ is the base per capita rate that underemployed people secure full employment per year, and $c$ is the effect of completing post-secondary education or vocational training on the underemployed to fully employed transition rate (see Wilkins, 2004, 2006). People in full employment become underemployed at age-specific rates $zE$, where $E$ is the fully employed population and $z$ is the per capita rate at which people transition from full employment to underemployment per year, so that the net flow from full employment to underemployment is $zE-ckM$. Fully employed and underemployed people leave the labour force (e.g., due to retirement, disability, parenting responsibilities) at constant per capita rates per year (separate age-specific rates are specified for people in full employment and those who are underemployed).

Net migration increases (or reduces) the fully employed population at age-specific rates $p_{i}I_{i}-q_{i}e_{i}P_{i}$, where $p_{i}$ and $q_{i}$ are the age-specific proportions of people in full employment among overseas arrivals and Australian residents, respectively, $I_{i}$ is total age-specific immigration per year, $e_{i}$ is the age-specific per capita emigration rate per year, and $P_{i}$ is the number of people in age group $i$ in the Australian population. The effects of migration on the unemployed and underemployed populations are modelled in the same way. Age-specific per capita mortality rates for people seeking employment are assumed to be 1.22 times those for people who are fully employed, underemployed, or not in the labour force (Sorlie and Rogot, 1990). Figure S11 presents participation and unemployment rate estimates derived from the system dynamics model and from labour force data published by the Australian Bureau of Statistics (2021).

*1.7. Mental health services sector*

Figure S12 shows a high-level map of the mental health services sector, which models the movement of patients through the mental health care system. People experiencing low or moderate to very high psychological distress engage with mental health services in one of two ways; they may perceive a need for mental health care and seek help (e.g., from a general practitioner or online services), or they may present to an emergency department (e.g., for self-harm) without having previously perceived a need for treatment. After engaging with mental health services, patients may recover following treatment, returning to the general population of people with low


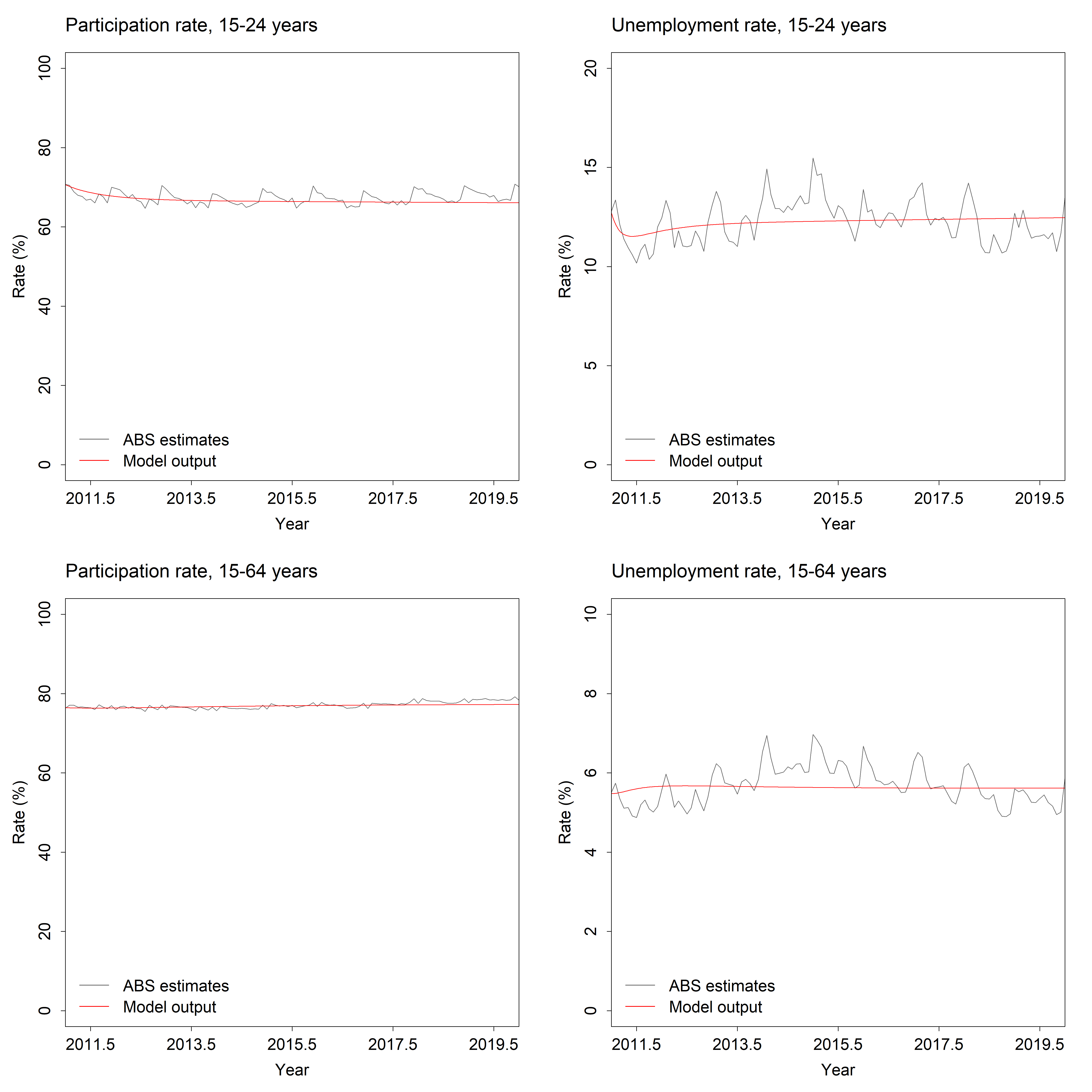


Figure S11. Participation and unemployment rate estimates derived from the system dynamics model and from Australian Bureau of Statistics (ABS; 2021) labour force data.

psychological distress and no perceived need for care, be treated but not recover, or disengage due to excessive waiting times (a result of insufficient services capacity) or because they are dissatisfied with the care they receive. Patients who are treated but do not recover return to perceiving a need for services and will eventually seek help again if they do not recover spontaneously; thus, people entering the mental health care system continue receiving treatment (modelled as individual service contacts; see below) until they recover, disengage, or die (mortality is captured in the model, but is not shown in figure S12). The principal components of the mental health services sector are described in detail below.


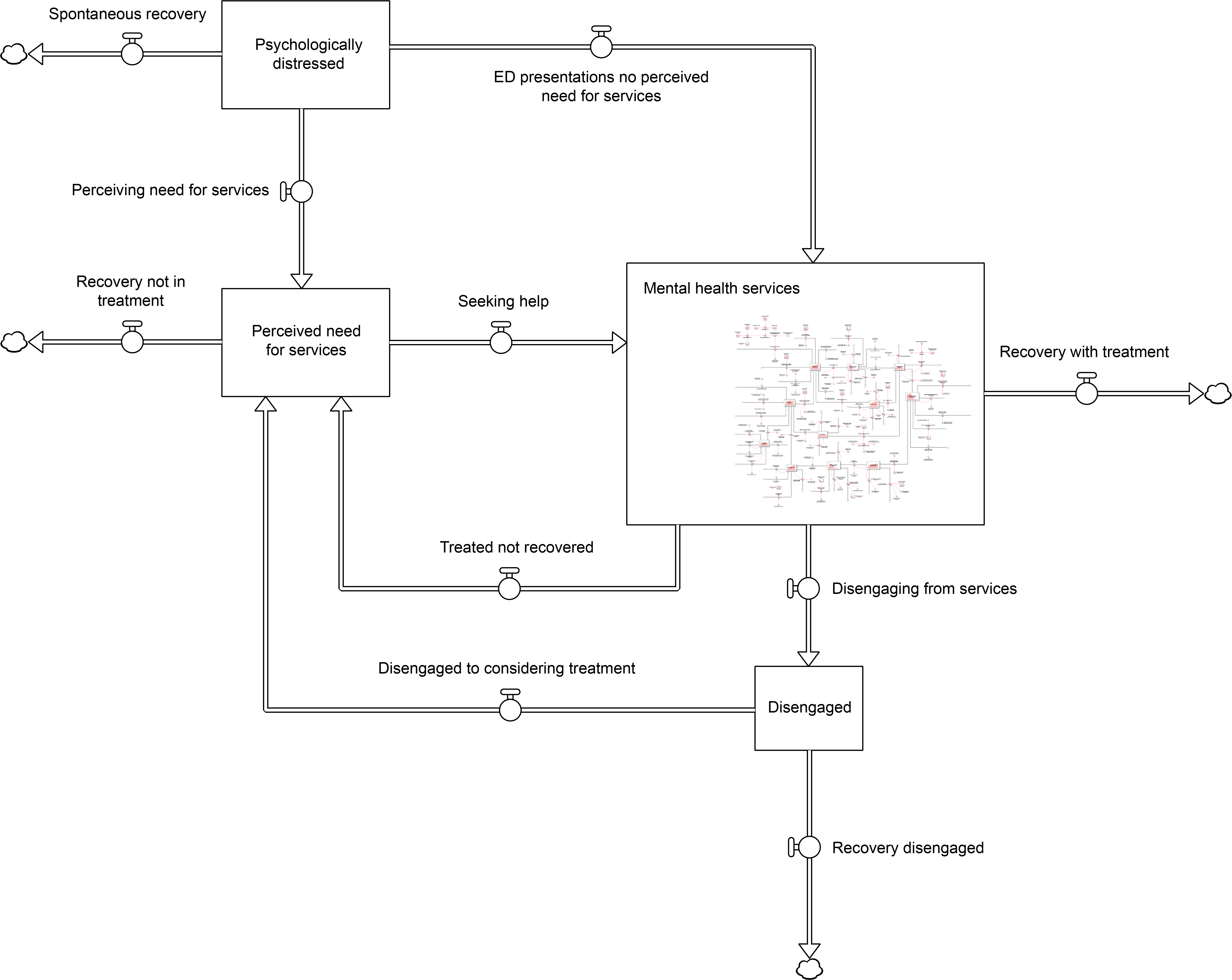


Figure S12. High-level map of the mental health services sector.

*Help seeking* — People experiencing low or moderate to very high psychological distress who are not currently considering engaging with mental health services perceive a need for care at rates equal to $p_{i}D_{i}$, where $D_{i}$ is the number of people with distress level $i$ not currently considering treatment and $p_{i}$ is the per capita rate that people with distress level $i$ perceive a need for care per year. The per capita rates $p_{i}$ are assumed to increase at a constant rate per year due to increasing public awareness of high-prevalence mental disorders and available treatment options. After perceiving a need for treatment, people engage with mental health services at per capita rates that depend on their age and state of psychological distress (Australian Bureau of Statistics, 2012). Recently treated patients who have not recovered or disengaged from services return to perceiving a need for care and may attend subsequent (planned or unplanned) appointments with a GP or community-based psychiatric services (i.e., psychiatrists and allied health services, hospital outpatient services), be admitted to a general or private hospital, commence online treatment, or present to an emergency department (e.g., for suicidal ideation); the arrayed stock of people perceiving a need for services (see figure S13) therefore contains a mix of prospective patients and patients already engaged with the mental health services system. These prospective and current patients may age, recover spontaneously, or transition from a state of low (mild) psychological distress to a state of moderate to very high distress, and are assumed to experience the same per capita mortality rates as similar-aged people with the same level of distress who are not considering treatment.

*GP and online services* — People with a perceived need for mental health services seek help from a GP at age-specific rates $\theta_{j}s_{i}P_{ij}$, where $P_{ij}$ is the number of people in age group $i$ with distress level $j$ perceiving a need for care (those in the arrayed stock labelled ‘Perceived need for services’ in figure S13), $s_{i}$ is the age-specific per capita rate at which people with low distress and a perceived need for care seek help from a GP, and the rate ratio $\theta_{j}$ is equal to 1 for people with low distress and less than 1 for people experiencing moderate to very high distress (i.e., people with moderate to very high psychological distress are assumed to seek help at a lower per capita rate than those with low distress, given a perceived need for care; see Australian Bureau of Statistics, 2012). The per capita rates $s_{i}$ are assumed to increase at a constant rate per year, increasing help seeking rates among people who perceive a need for services. Prior to receiving care, patients seeking help from a GP or referred to GP services after completing hospital inpatient care wait for a varying period of time that depends on services capacity and the total number of patients waiting for care. GP services capacity, i.e., the number of mental health-related GP consultations that can be provided per year, is assumed to increase at a constant rate per year, estimated from Medicare Benefits Schedule (MBS) claims data for the period 2011−2019 (see figure S14).

Mental health-related GP consultations are represented as a stock (arrayed by age group and distress level) with outflows corresponding to treatment completion, referral to other services (including psychiatrist and allied health services, community mental health care services, and online services), and disengagement resulting from dissatisfaction with the care provided. Age-specific recovery rates among patients completing treatment are equal to $v\gamma_{j}rT_{ij}$, where $T_{ij}$ is the number of patients in age group $i$ with distress level $j$ completing treatment per year, $v$ is the proportion of patients completing treatment who receive psychological therapy, $r$ is the proportion of patients with low (or mild) distress who recover after receiving psychological therapy, and the recovery rate ratio $\gamma_{j}$ is equal to 1 for mildly distressed patients and less than 1 for patients experiencing moderate to very high psychological distress (i.e., psychological therapy provided by a GP is assumed to be less effective for moderately to highly distressed patients than for mildly distressed patients; see Cuijpers et al., 2009). Patients completing treatment who do not recover return to the stocks of people who perceive a need for care.


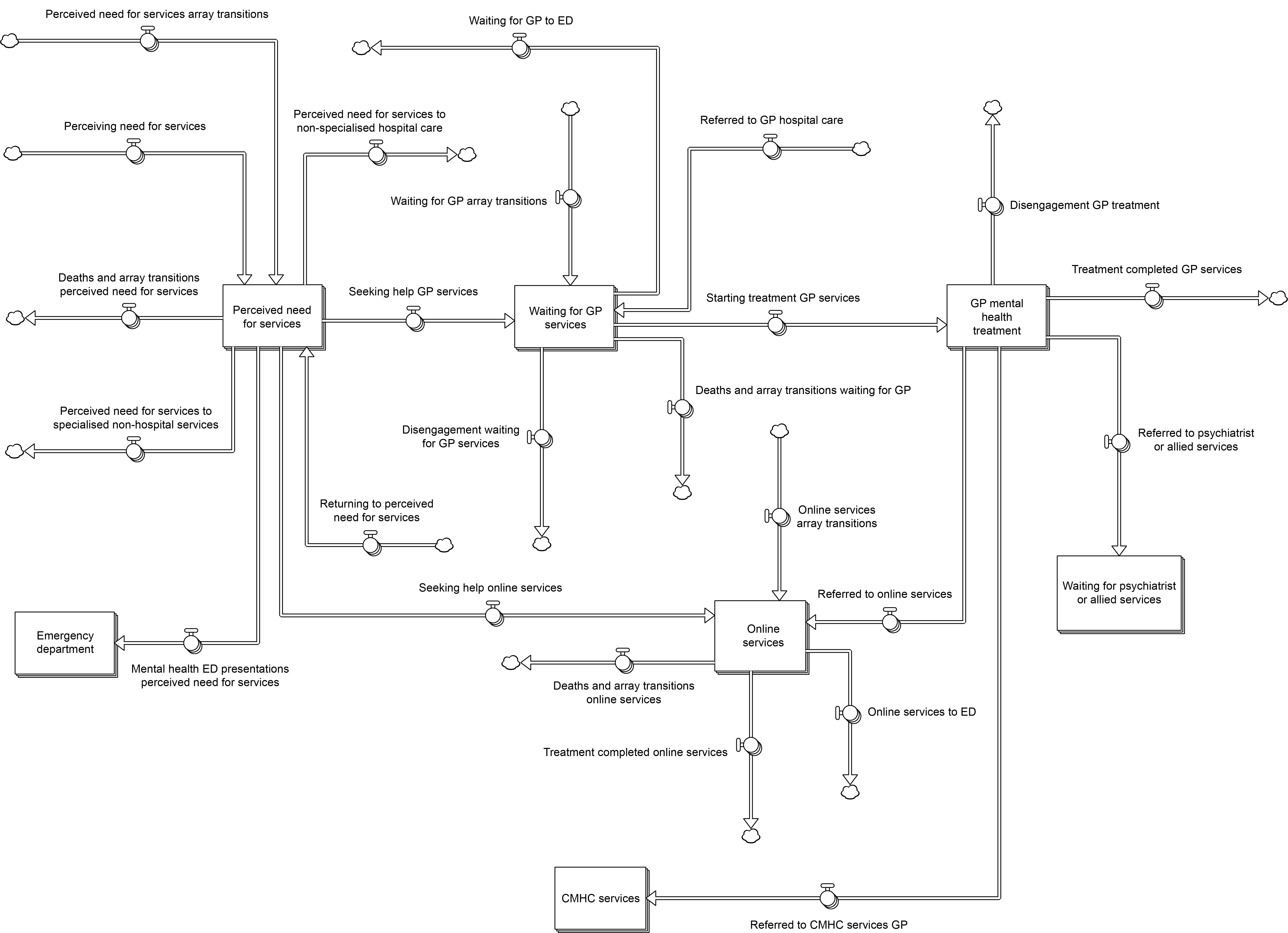


Figure S13. Stock and flow structure of the help-seeking, general practitioner (GP) services, and online services components of the mental health services sector.


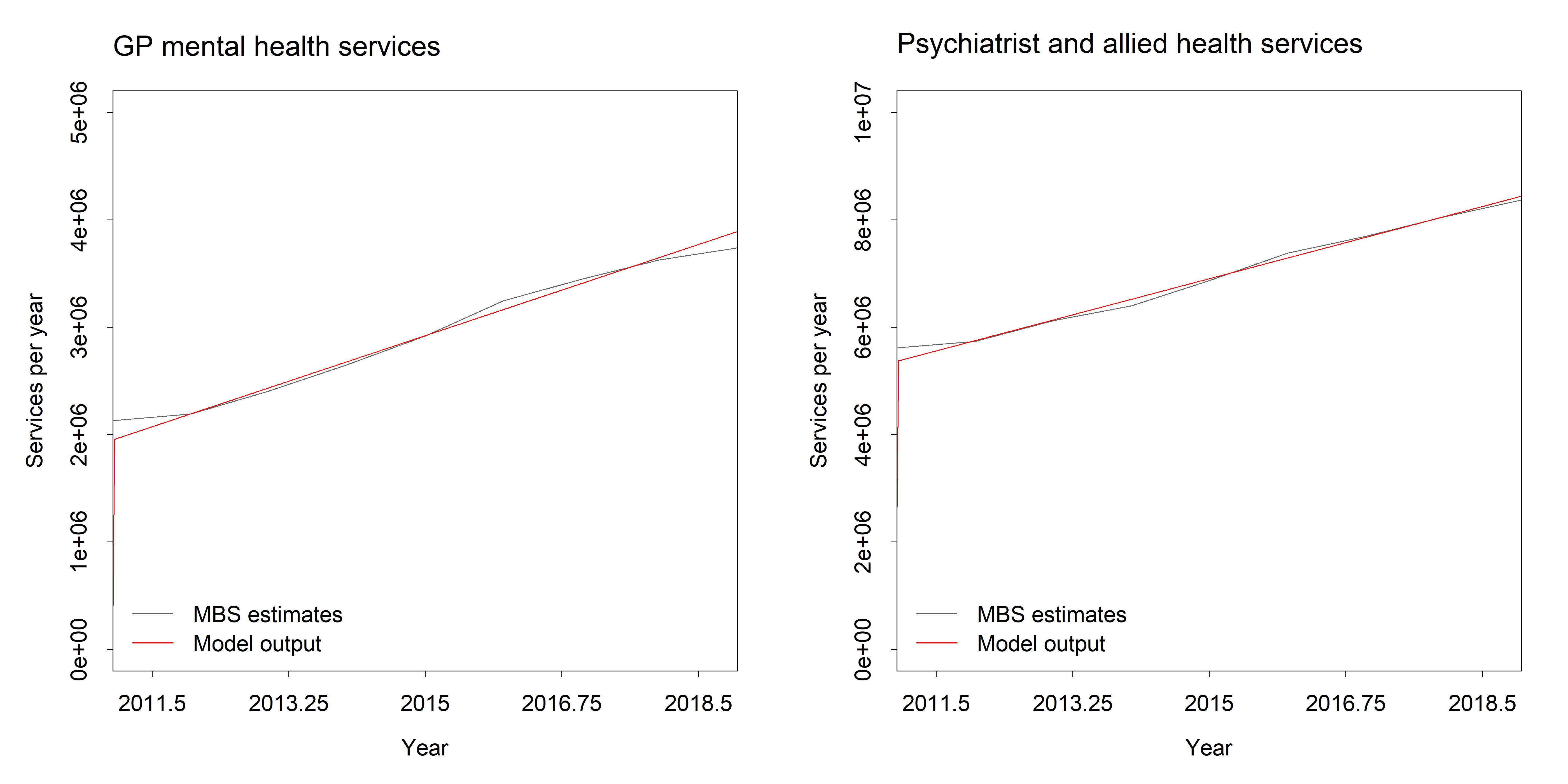


Figure S14. General practitioner (GP) and psychiatrist and allied health services usage rates derived from the system dynamics model and from Medicare Benefits Schedule (MBS) data (available at: https://www.aihw.gov.au/reports/mental-health-services/mental-health-services-in-australia/data).

Age-specific treatment commencement rates for online (self-help) services are equal to $h_{j}P_{ij}+uG_{ij}$, where $P_{ij}$ is the number of people in age group $i$ with psychological distress level $j$ who perceive a need for services (i.e., those in the arrayed stock labelled ‘Perceived need for services’ in figure S13), $h_{j}$ is the per capita rate that people with distress level $j$ and a perceived need for care access online services per year, $G_{ij}$ is the GP services provision rate (i.e., the number of patients in age group $i$ with psychological distress level $j$ attending a GP consultation per year), and $u$ is the fraction of patients visiting a GP for a mental health issue referred to online services. Prior to completing treatment, people accessing online services may recover spontaneously, present to an emergency department, or die (see figure S13); treatment completion rates are equal to ${O_{ij}}/d$, where $O_{ij}$ is the number of people in age group $i$ with distress level $j$ engaged in online treatment and $d$ is the mean duration of online treatment programs (assumed to be 6 weeks, or 0.115 years; Christensen et al., 2004). People completing online treatment programs recover at rates equal to $\eta_{j}z{O_{ij}}/d$, where $z$ is the fraction of people with low (or mild) psychological distress completing treatment who recover and the rate ratio $\eta_{j}$ is equal to 1 for mildly distressed people and significantly less than 1 for people with moderate to very high distress (i.e., online services are assumed to be more effective for people with a low level of distress than for people with moderate to very high levels of distress).

*Psychiatrist and allied health services* — Figure S15 presents the structure of the psychiatrist and allied health services component of the mental health services sector. Prior to receiving treatment, patients referred to a psychiatrist or allied health services by a GP or after completing hospital inpatient care wait for a period of time that depends on services capacity and the total number of patients waiting for care. The arrayed stock of people waiting for treatment also contains patients currently engaged with specialised services who have planned (follow-up) appointments (these patients and patients referred to services after receiving hospital care enter via the flow labelled ‘Additional psychiatrist or allied health services’; see figure S15). Services capacity, i.e., the number of psychiatrist and allied health services that can be provided per year, increases at a constant rate per year, estimated from MBS claims data for the period 2011−2019 (see figure S14). Patients receiving treatment are referred to psychiatric hospital services, disengage from the mental health services system due to dissatisfaction with the care received, recover, or return to perceiving a need for care (these patients flow back into the arrayed stock labelled ‘Perceived need for services’; see figure S13). Recovery rates are equal to $rT_{ij}$, where $T_{ij}$ is the number of patients in age group $i$ with psychological distress level $j$ who complete treatment per year and $r$ is the fraction of patients recovering after receiving specialised psychiatric care. Psychiatrist and allied health services usage rates derived from the model and from MBS claims data are presented in figure S14.

*Hospital services* — Mental health-related public hospital services captured in the model include psychiatric and non-specialised inpatient care, outpatient care delivered by community mental health care (CMHC) teams, and emergency department attendances (figure S16). Emergency department (ED) presentation rates are equal to $\theta_{j}e_{i}C_{ij}+\theta_{j}f_{i}N_{ij}$, where $C_{ij}$ is the number of people in age group $i$ with psychological distress level $j$ perceiving a need for care (including all patients waiting for care or engaged in online treatment and those who have disengaged from services; see figures S13, S15, S16, S18, S19), $N_{ij}$ is the number of people in age group $i$ with distress level $j$ who do not perceive a need for services, $e_{i}$ and $f_{i}$ are age-specific per capita ED presentation rates for mildly distressed people with and without a perceived need for care, respectively, and the rate ratio $\theta_{j}$ is equal to 1 for people with low (mild) psychological distress and more than 1 for people experiencing moderate to very high distress (i.e., people in a state of moderate to very high distress are assumed to present to EDs at a higher per capita rate than those with a low level of distress). People presenting to an ED may be admitted to a psychiatric or general hospital ward, referred to CMHC services, or discharged to the community. A fraction of patients perceiving a need for care who are discharged to the community disengage from services due to dissatisfaction with the care provided.


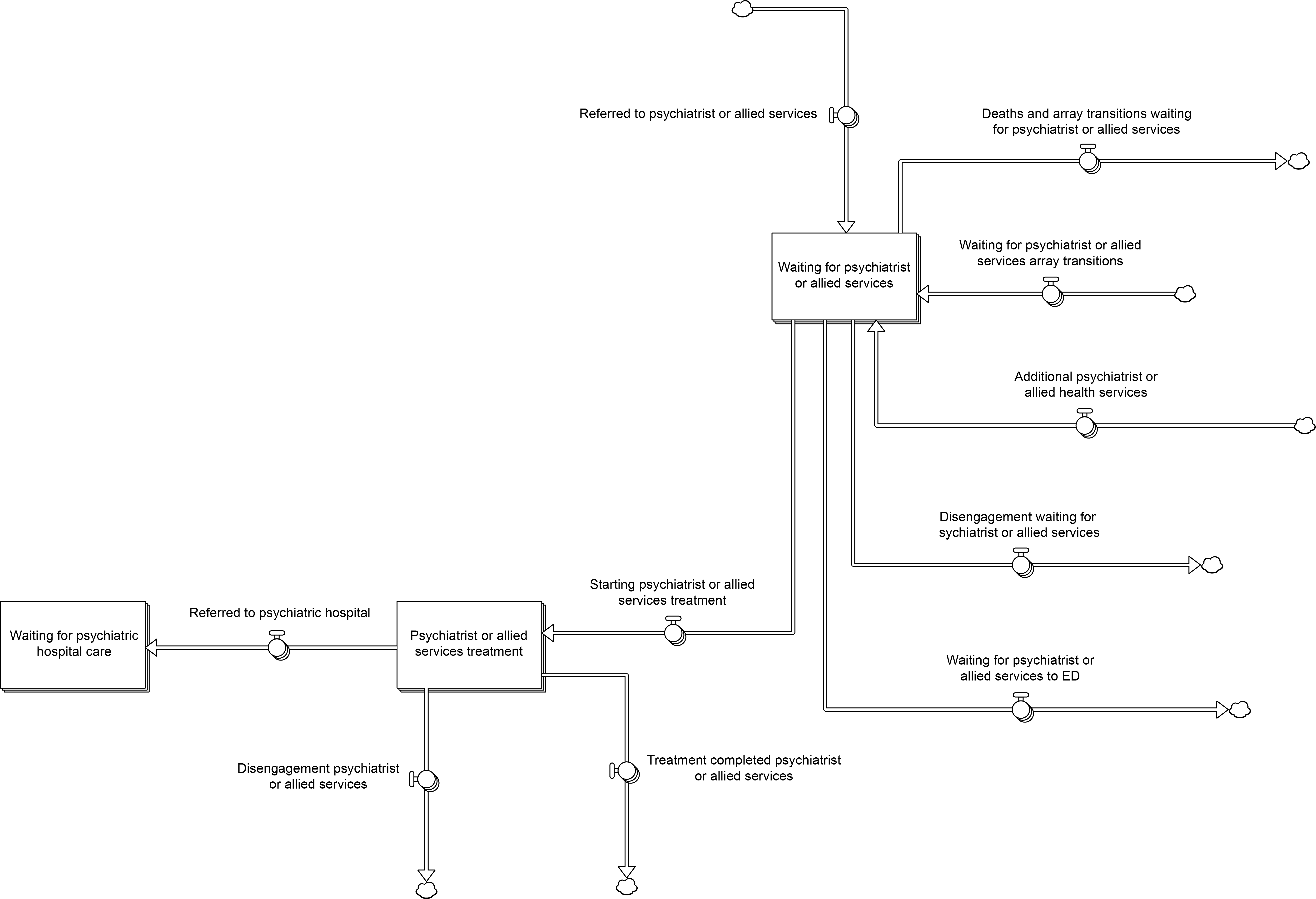


Figure S15. Stock and flow structure of the psychiatrist and allied health services component of the mental health services sector.


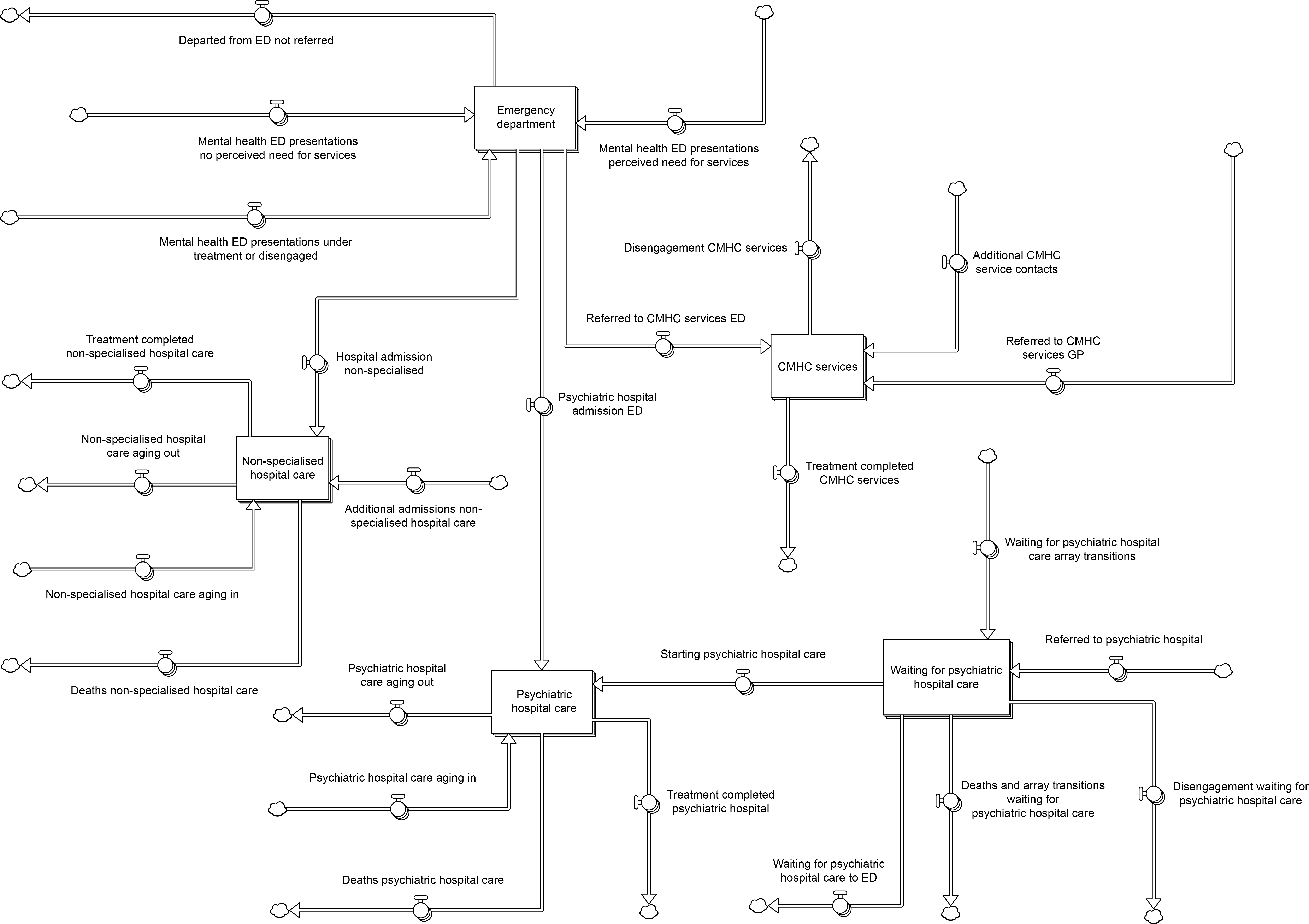


Figure S16. Stock and flow structure of the hospital services component of the mental health services sector.

Psychiatric and general hospital admission rates are constrained by hospital capacity (i.e., the total numbers of patients that can be admitted annually), which is assumed to increase linearly over the simulation period; capacity increase rates per year were estimated from mental health-related hospital separations data published by the Australian Institute of Health and Welfare (2019; see figure S17). Psychiatrists refer patients to psychiatric hospital care at rates $q_{i}M_{ij}$, where $M_{ij}$ is the number of patients in age group $i$ with distress level $j$ receiving psychiatrist and allied health services per year and $q_{i}$ is the age-specific proportion of patients receiving psychiatrist and allied health services referred to psychiatric hospital care. Prior to being admitted, patients referred to a psychiatric hospital wait for a period of time that depends on hospital capacity, the total number of referred patients waiting for care, and the rate at which patients are being admitted via EDs (since available capacity declines as the ED-related admission rate increases). People with a perceived need for services are admitted for general (non-specialised) hospital care without presenting to an ED (e.g., for allied health interventions) at constant per capita rates that depend on age and distress level (the flow labelled ‘Additional admissions non-specialised hospital care’ in figure S16). A proportion of patients discharged from psychiatric or general hospital care disengage from services due to dissatisfaction with the care provided; patients who do not disengage from care are referred to a GP, psychiatrist and allied health services, or CMHC services for follow-up care.

Age-specific community mental health care (CMHC, or hospital outpatient) services provision rates are equal to $\eta_{j}h_{i}G_{ij}+pE_{ij}+S_{ij}+A_{ij}+k_{j}u_{i}P_{ij}$, where $G_{ij}$ is the number of patients in age group $i$ with distress level $j$ visiting a GP per year, $h_{i}$ is the proportion of mildly distressed patients in age group $i$ visiting a GP who are referred to CMHC services, $E_{ij}$ is the number of people presenting to an ED per year not admitted to hospital, $p$ is the proportion of patients discharged from an ED referred to CMHC services, $S_{ij}$ and $A_{ij}$ are, respectively, the numbers of patients discharged from psychiatric and general hospital inpatient care per year referred to CMHC services, $P_{ij}$ is the number of people with a perceived need for care (those in the arrayed stock labelled ‘Perceived need for services’; figure S13), $u_{i}$ is the per capita CMHC services contact rate among people with a perceived need for care (e.g., for follow-up appointments), and the rate ratios $\eta_{j}$ and $k_{j}$ are equal to 1 for patients with low psychological distress and more than 1 for patients with moderate to very high psychological distress. Patients receiving CMHC services recover, return to the stocks of people perceiving a need for services, or disengage from the mental health care system due to dissatisfaction with the care provided (figure S16). The per-service recovery rate for patients who do not disengage from treatment is calculated as ${rK}/T$, where $K$ is the total number of CMHC services that can be provided per year while maintaining the base (or reference) per-service recovery rate $r$ and $T$ is the current CMHC services provision rate; note that as the current services provision rate, $T$, increases relative to the reference capacity $K$, the per-service recovery rate, ${rK}/T$, declines (due to increased pressure on services). CMHC services capacity (i.e., $K$) is assumed to increase at a constant rate per year, estimated from services usage data published by the Australian Institute of Health and Welfare (2019; figure S17).


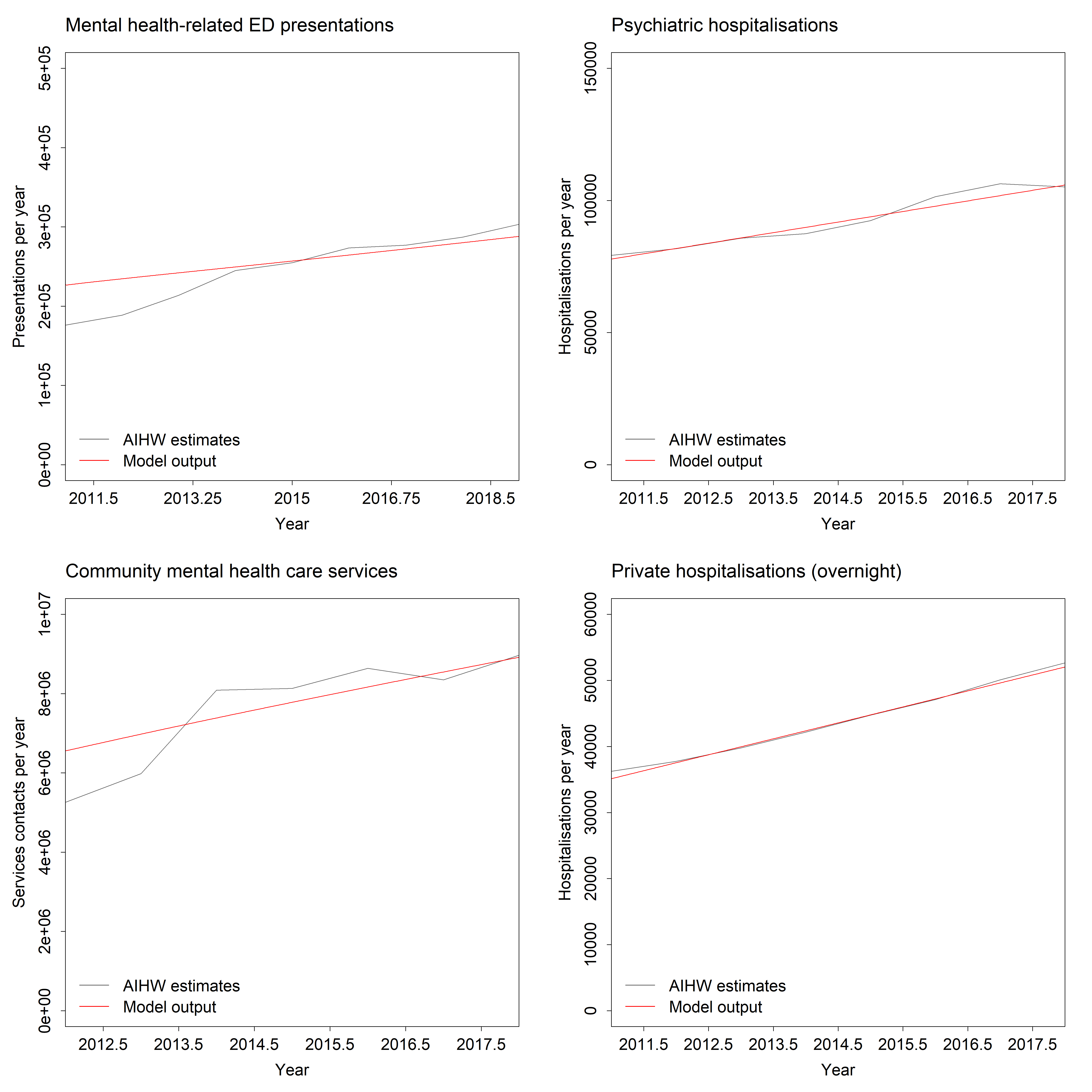


Figure S17. Hospital services usage rates derived from the system dynamics model and from data published by the Australian Institute of Health and Welfare (AIHW; available at: https://www.aihw.gov.au/reports/mental-health-services/mental-health-services-in-australia/data).

*Private mental health services* — The structure of the private services component of the mental health services sector, which captures overnight inpatient care provided in private hospitals and community-based private mental health services (including hospital outpatient and same-day inpatient care; see Australian Institute of Health and Welfare, 2019), is shown in figure S18. Private mental health services are assumed to operate independently of the government financed mental health services described above (there are no direct referral pathways connecting public and private services), although individual patients may access both private and public services at different times. Patients are referred to private hospitals for overnight inpatient care at age-specific rates $s_{i}P_{i}$, where $P_{i}$ is the number of people in age group $i$ perceiving a need for care (those in the arrayed stock labelled ‘Perceived need for services’ in figure S13) and $s_{i}$ is the age-specific per capita referral rate per year. Prior to being admitted, patients referred to a private hospital wait for a period of time that depends on hospital capacity and the total number of patients waiting for treatment. Private hospital capacity, i.e., the number of patients that can be admitted annually, is assumed to increase at a constant rate per year, estimated from data published by the Australian Institute of Health and Welfare (2019; see figure S17). A proportion of patients discharged following overnight inpatient care disengage from the services system due to dissatisfaction with the care provided; patients who do not disengage are referred to community-based private mental health services for follow-up care.

Age-specific rates of referral to community-based private services are equal to $u_{i}P_{i}+\left( 1-d \right)H_{i}$, where $P_{i}$ is the number of people in age group $i$ perceiving a need for care (i.e., those in the arrayed stock labelled ‘Perceived need for services’ in figure S13), $u_{i}$ is the age-specific per capita referral rate per year, $H_{i}$ is the number of private hospital patients in age group $i$ discharged from overnight inpatient care per year, and $d$ is the proportion of discharged patients disengaging from mental health services due to dissatisfaction with the care provided. Prior to receiving treatment, patients referred to community-based private services wait for a period of time that depends on services capacity and the total number of referred patients waiting for care. Services capacity, i.e., the total number of community-based services that can be delivered per year, is assumed to increase at a constant rate per year, estimated from data published by the Australian Institute of Health and Welfare (2019). Patients receiving treatment disengage from the services system due to dissatisfaction with the care provided, recover, or return to perceiving a need for care (these patients flow back into the arrayed stock labelled ‘Perceived need for services’; figure S13). Recovery rates are equal to $rT_{ij}$, where $T_{ij}$ is the number of patients in age group $i$ with psychological distress level $j$ completing treatment per year and $r$ is the fraction of patients recovering after receiving specialised psychiatric care.


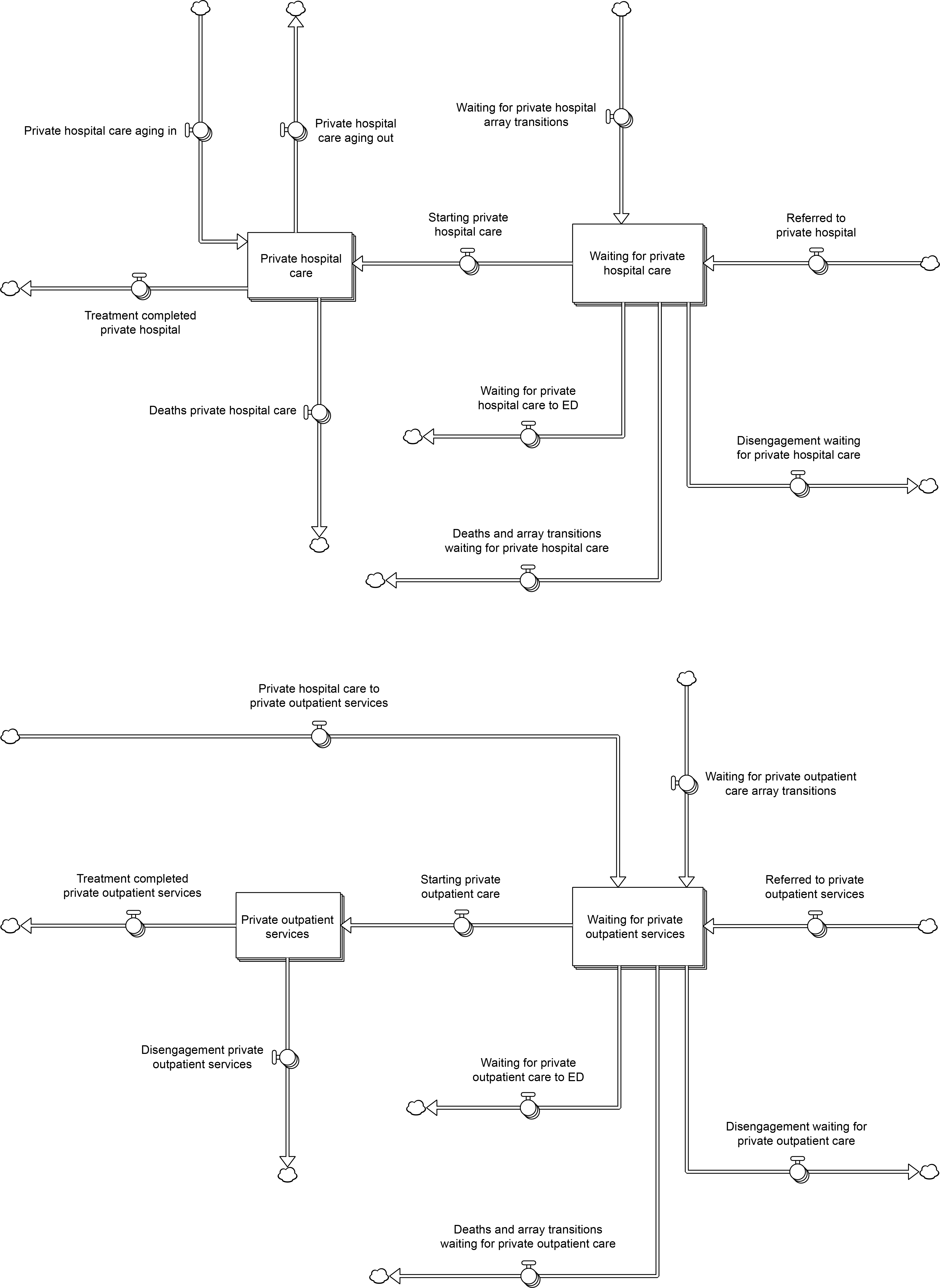


Figure S18. Stock and flow structure of the private mental health services component of the mental health services sector.

*Disengagement* — Patients waiting for a GP, psychiatrist and allied health services, psychiatric hospital care, or private services are assumed to disengage from the mental health care system at a constant per capita rate per year (estimated from data reported in Tyrer et al., 1995). The total disengagement rate therefore increases whenever the demand for mental health services exceeds services capacity, as the number of patients waiting for care continues to increase while patients are being referred to services at a higher rate than they can be treated. Patients receiving treatment also disengage from services due to dissatisfaction with the care provided (figures S13, S15, S16, S18). Disengagement is assumed to increase the age-specific per capita rates at which people with low psychological distress transition to a state of moderate to very high psychological distress, due to a loss of hope that effective treatment is available, or trauma associated with unsatisfactory care (see Australian Bureau of Statistics, 2012). Patients who have disengaged from the services system return to the arrayed stock of people perceiving a need for care (i.e., they consider engaging with services again) at a constant per capita rate per year (figure S19).


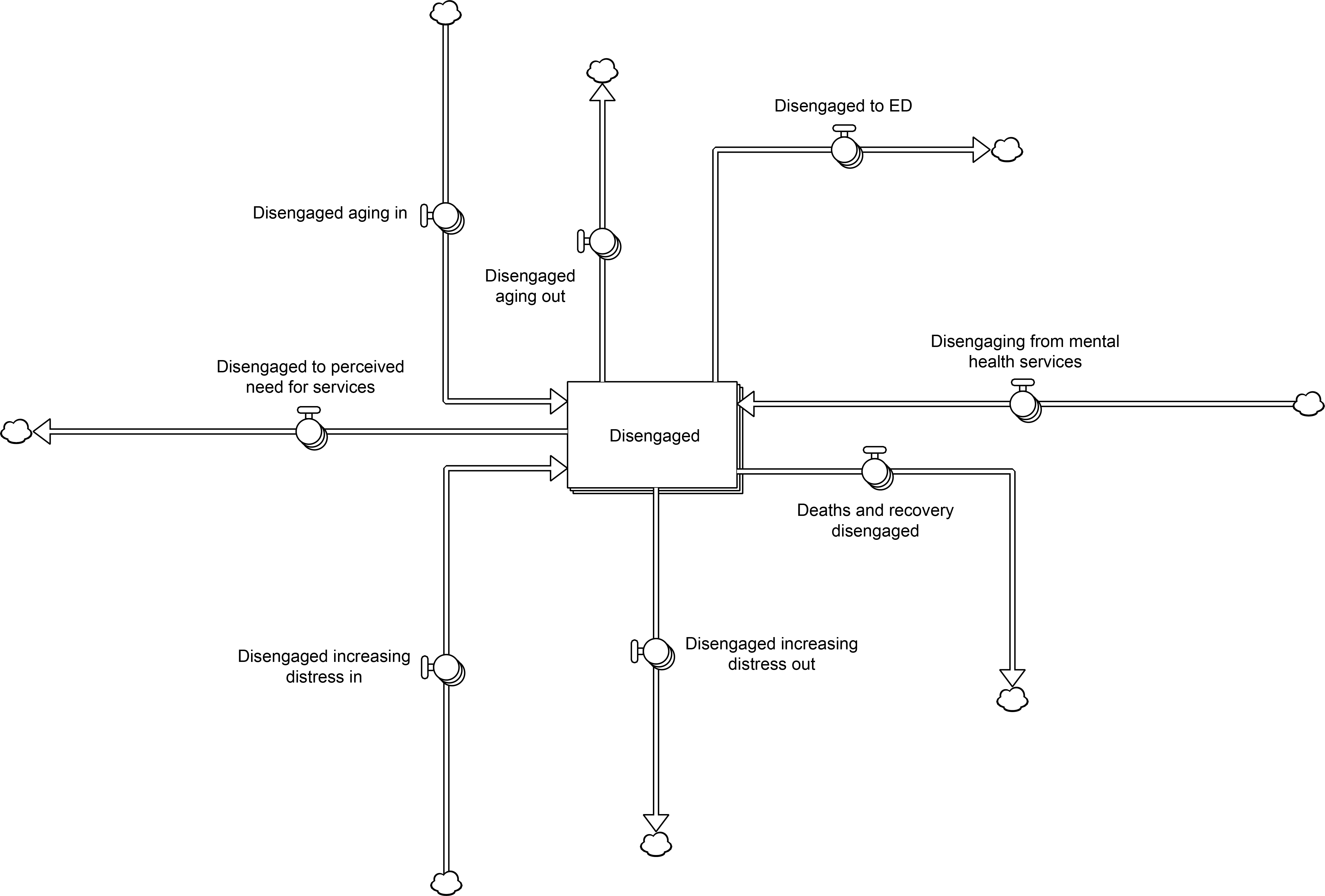


Figure S19. Stock and flow structure of the disengagement component of the mental health services sector.

*1.8. Suicidal behaviour sector*

Figure S20 presents the structure of the suicidal behaviour sector, which captures self-harm hospitalisations and suicide deaths (note that we equate suicide attempts with intentional self-harm hospital admissions due to data availability constraints; see the Discussion section of the paper). Age-specific suicide attempt rates are calculated as $s_{i}M_{i}+\theta s_{i}H_{i}$, where $M_{i}$ and $H_{i}$ are the numbers of people in age group $i$ experiencing low (mild) psychological distress and moderate to very high psychological distress, respectively, $s_{i}$ is the per capita suicide attempt rate for mildly distressed people in age group $i$, and the suicide attempt rate ratio $\theta$ is assumed to be substantially greater than 1 (i.e., the per capita attempt rate for people with mild distress is assumed to be lower than that for people experiencing moderate to very high distress; Chamberlain et al., 2009). The number of suicide deaths per year is calculated as $\lambda a$, where $a$ is the suicide attempt rate and $\lambda$ is attempt lethality (i.e., the proportion of suicide attempts that are fatal). Intentional self-harm hospitalisation and suicide death rate estimates derived from the system dynamics model and from data published online by the Australian Institute of Health and Welfare (available at: https://www.aihw.gov.au/suicide-self-harm-monitoring/data) are presented in figure S21.


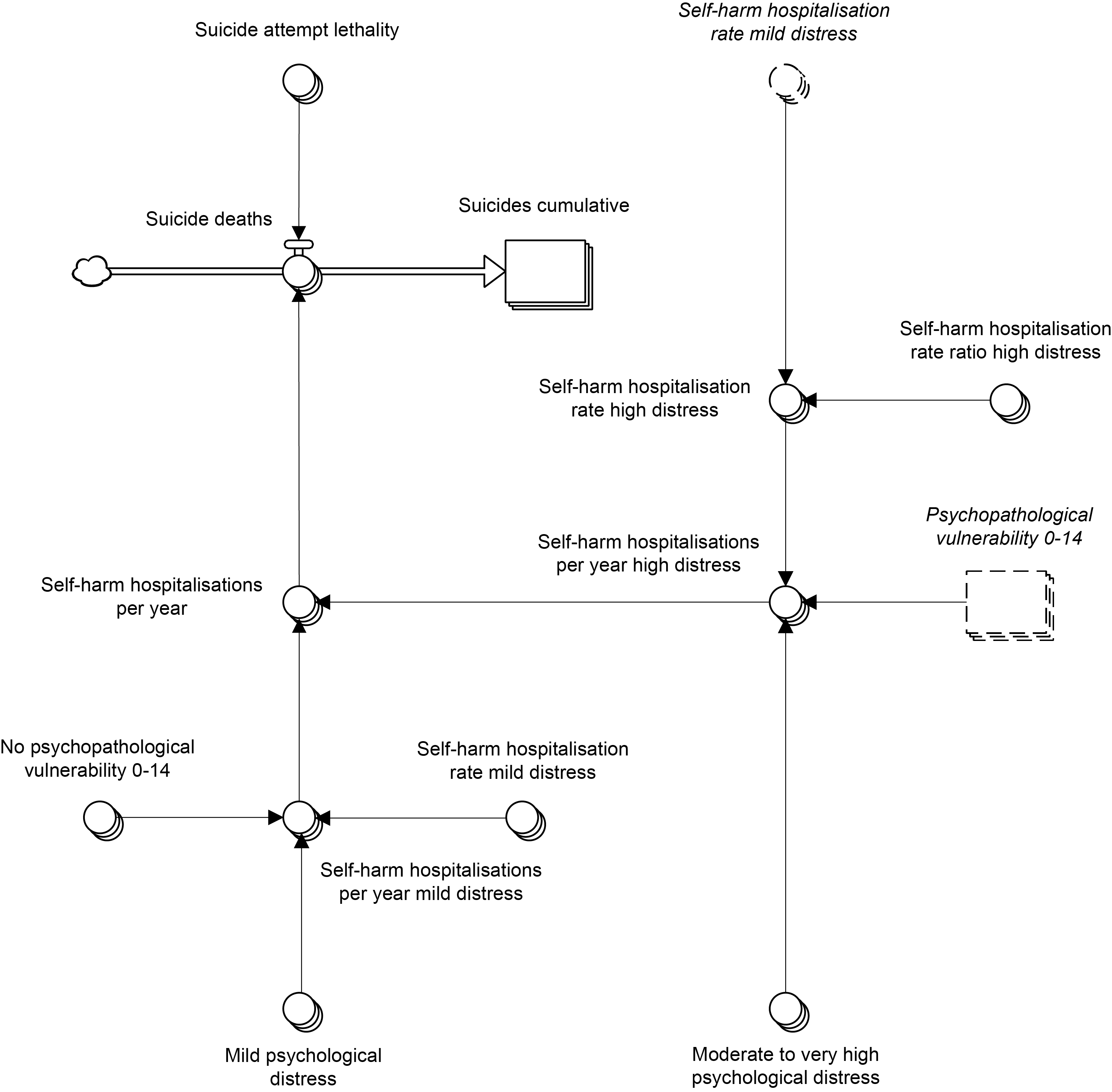


Figure S20. Structure of the suicidal behaviour sector.


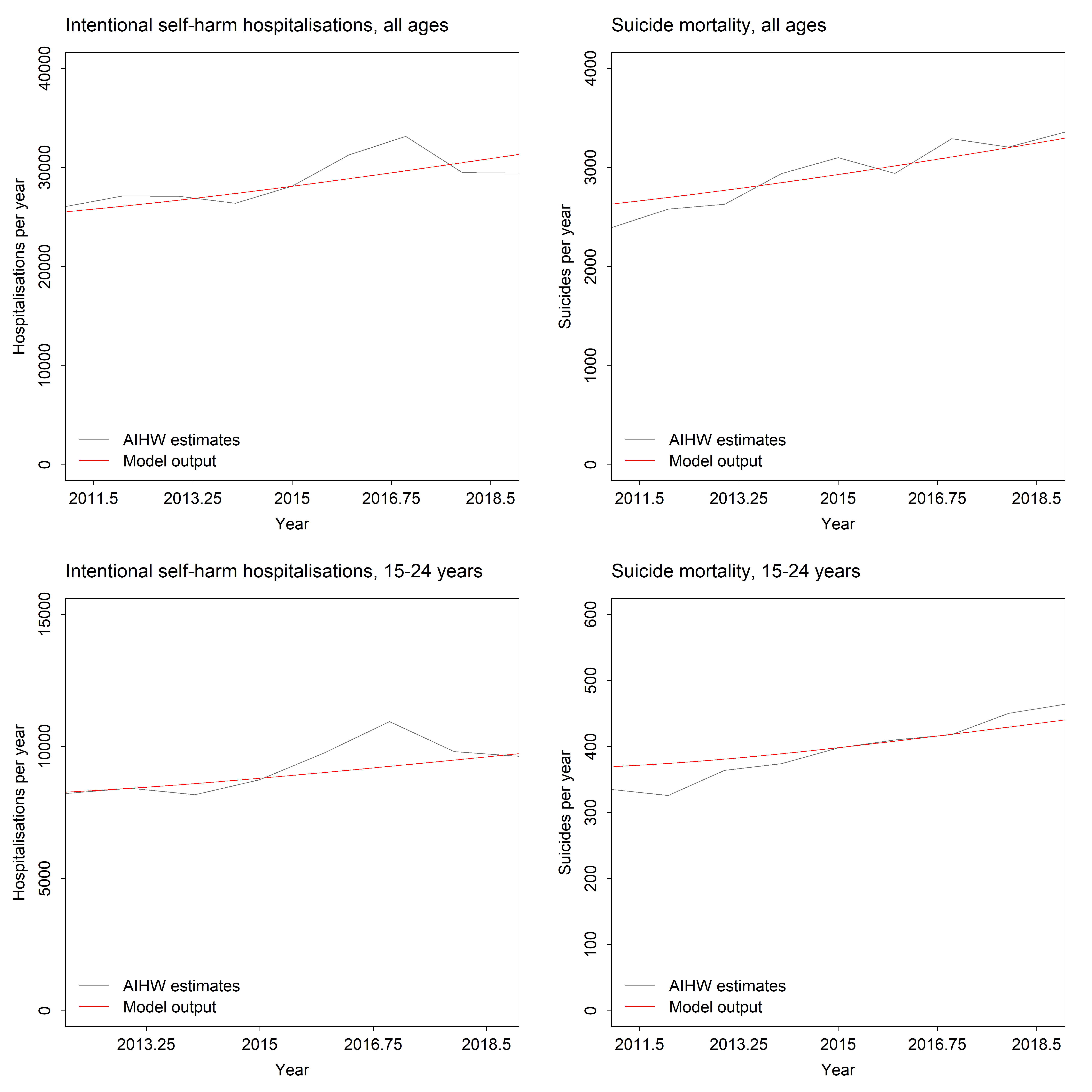


Figure S21. Intentional self-harm hospitalisation and suicide mortality rate estimates derived from the system dynamics model and from data published online by the Australian Institute of Health and Welfare (available at: https://www.aihw.gov.au/suicide-self-harm-monitoring/data)

References

Australian Bureau of Statistics, 2012. Information paper. Use of the Kessler psychological distress scale in ABS health surveys, Australia, 2007−08. Cat. no. 4817.0.55.001. Australian Bureau of Statistics, Canberra.

Australian Bureau of Statistics, 2018. National Health Survey: first results, 2017−18. Cat. no. 4364.0.55.001. Australian Bureau of Statistics, Canberra.

Australian Bureau of Statistics, 2019. Australian demographic statistics, Jun 2019. Cat. no. 3101.0. Australian Bureau of Statistics, Canberra.

Australian Bureau of Statistics, 2020. Education and work, Australia, May 2020. Cat. no. 6227.0. Australian Bureau of Statistics, Canberra.

Australian Bureau of Statistics, 2021. Labour force, Australia, Mar 2021. Cat. no. 6202.0. Australian Bureau of Statistics, Canberra.

Australian Institute of Health and Welfare, 2019. Mental health services − in brief 2019. Cat. no. HSE 228. Australian Institute of Health and Welfare, Canberra.

Backlund, E., Sorlie, P. D., Johnson, N. J., 1999. A comparison of the relationships of education and income with mortality: the national longitudinal mortality study. Soc. Sci. Med. 49, 1373−1384.

Chamberlain, P., Goldney, R., Delfabbro, P., Gill, T., Dal Grande, L., 2009. Suicidal ideation. The clinical utility of the K10. Crisis 30, 39−42.

Christensen, H., Griffiths, K. M., Jorm, A. F., 2004. Delivering interventions for depression by using the internet: randomised controlled trial. Br. Med. J. 328, 265.

Cuijpers, P., van Straten, A., van Schaik A., Andersson, G., 2009. Psychological treatment of depression in primary care: a meta-analysis. Br. J. Gen. Pract., doi: 10.3399/bjgp09X395139.

Dean, K., Green, M. J., Laurens, K. R., Kariuki, M., Tzoumakis, S., Sprague, T., Lenroot, R., Carr, V. J., 2018. The impact of parental mental illness across the full diagnostic spectrum on externalising and internalising vulnerabilities in young offspring. Psychol. Med. 48, 2257−2263.

Dooley, D., Prause, J., Ham-Rowbottom, K. A., 2000. Underemployment and depression: longitudinal relationships. J. Health Soc. Behav. 41, 421−436.

Frijters, P., Johnston, D. W., Shields, M. A., 2014. The effect of mental health on employment: evidence from Australian panel data. Health Econ. 23, 1058−1071.

Green, M. J., Tzoumakis, S., Laurens, K. R., Dean, K., Kariuki, M., Harris, F., Brinkman, S. A., Carr, V. J., 2019. Early developmental risk for subsequent childhood mental disorders in an Australian population cohort. Aust. N. Z. J. Psychiatry 53, 304−315.

Lee, S., Tsang, A., Breslau, J., Aguilar-Gaxiola, S., Angermeyer, M., Borges, G., Bromet, E., Bruffaerts, R., de Girolamo, G., Fayyad, J., Gureje, O., Haro, J. M., Kawakami, N., Levinson, D., Oakley Browne, M. A., Ormel, J., Posada-Villa, J., Williams, D. R., Kessler, R. C., 2009. Mental disorders and termination of education in high-income and low- and middle-income countries: epidemiological study. Br. J. Psychiatry 194, 411−417.

Mitchell, W., Wray, L. R., Watts, M., 2019. Macroeconomics. Red Globe Press, London.

Russ, T. C., Stamatakis, E., Hamer, M., Starr, J. M., Kivimäki, M., Batty, G. D., 2012. Association between psychological distress and mortality: individual participant pooled analysis of 10 prospective cohort studies. Br. Med. J. 345, e4933.

Sorlie, P. D., Rogot, E., 1990. Mortality by employment status in the National Longitudinal Mortality Study. Am. J. Epidemiol. 132, 983−992.

Tyrer, P., Morgan, J., Van Horn, E., Jayakody, M., Evans, K., Brummell, R., White, T., Baldwin, D., Harrison-Read, P., Johnson, T., 1995. A randomised controlled study of close monitoring of vulnerable psychiatric patients. Lancet 345, 756−759.

Wilkins, R., 2004. The extent and consequences of underemployment in Australia. Melbourne Institute working paper no. 16/04. The University of Melbourne, Melbourne.

Wilkins, R., 2006. Personal and job characteristics associated with underemployment. Aust. J. Labour Econ. 9, 371−393.

Supplementary appendix 2

Modelling the impact of COVID-19

The demographic, economic, health services, and psychological impacts of the continuing COVID-19 pandemic (see, e.g., Atkinson et al., 2020; Brooks et al., 2020; Charles-Edwards et al., 2020; Moreno et al., 2020) were modelled as abrupt changes in multiple flows directly affected by infection control measures (lockdowns, social distancing, international and interstate travel restrictions), including: 1) a decrease in the number of people arriving from overseas per year; 2) increases in the per capita rates at which people transition from employment (including underemployment) to unemployment and from full employment to underemployment; 3) reductions in per capita rates of non-acute mental health services provision (including general practitioner services, psychiatrist and allied health services, public hospital outpatient services, and private mental health services); and 4) an increase in the incidence of moderate to very high psychological distress resulting from social dislocation unrelated to job loss (e.g., working from home, not participating in recreational activities, restricted social gatherings) and anxiety about potential unemployment (Dooley et al., 1988). Parameter values determining the scale and duration of these direct effects (which begin on 1 March 2020 and decay over time) were estimated via constrained optimisation, implemented in Stella Architect ver. 1.9.4, using population projections for 2025 and 2030 reported by Charles-Edwards et al. (2020) and data on labour force status, psychological distress, and Medicare-subsidised mental health services usage (general practitioner, psychiatrist, and allied health services) available from the Australian Bureau of Statistics (2022a, b) and the Australian Government Department of Health (http://medicarestatistics.humanservices.gov.au/statistics/mbs_item.jsp) (see figure S22).

National survey data for the period after mid-March 2020 (when stringent infection control measures were first implemented in Australia) indicate that after increasing significantly in the initial months of the pandemic, the prevalence of psychological distress fell relatively rapidly as public health restrictions eased and unemployment and underemployment declined towards pre-pandemic levels in late 2020 and early 2021 (Biddle et al., 2020; Australian Bureau of Statistics, 2022b; Biddle and Gray, 2021). This suggests that a subset of people experiencing increased psychological distress due to COVID-19-related social and economic disruption did not develop persistent mental disorders and had the capacity to recover quickly when the direct impacts of the pandemic subsided. As a means of accommodating this distressed but resilient subpopulation, we allow the per capita spontaneous recovery rate (see section 1.3) to increase as the prevalence of psychological distress increases above that observed immediately prior to the start of the pandemic (i.e., from 1 March 2020). During early-mid 2020, decreasing social connectedness and increasing unemployment and underemployment drive an increase in the prevalence of moderate to very high psychological distress via increased distress incidence; however, because many people becoming psychologically distressed have the capacity to recover more rapidly than those with persistent mental disorders, the per capita spontaneous recovery rate also increases. As the direct social and economic effects of the pandemic abate, the incidence of moderate to very high psychological distress declines, and the higher per capita recovery rate results in a relatively rapid decrease in distress prevalence, consistent with the empirical data (see figure S22; note that as distress prevalence declines, the per capita spontaneous recovery rate also declines as acutely distressed people without persistent disorders recover).

Among people becoming psychologically distressed as a result of the pandemic, the risk of intentional self-harm and suicide is assumed to increase gradually (rather than immediately), consistent with evidence for a significant association between unemployment duration and suicidal behaviour (Milner et al., 2013) and a delay in the impact of economic recessions on suicide mortality (Garcy and Vågerö, 2013). From 1 March 2020, intentional self-harm hospitalisation rates (per year) for people aged 15 years and above are calculated as $s_{i}M_{i}+\theta s_{i}\left( p/q \right)H_{i}+f\left( t \right)s_{i}\left( 1-p/q \right)H_{i}$, where $M_{i}$ and $H_{i}$ are the numbers of people in age group $i$ experiencing low (mild) psychological distress and moderate to very high psychological distress, respectively, $s_{i}$ is the per capita suicide attempt rate per year for mildly distressed people in age group $i$, $\theta$ is the suicide attempt rate ratio among moderately to highly distressed people who were distressed before the pandemic began or who would have become distressed if the pandemic had not occurred, $q$ is the prevalence of moderate to very high psychological distress, $p$ is the prevalence of moderate to very high psychological distress immediately prior to 1 March 2020 (i.e., before the implementation of infection control measures), and $f\left( t \right)$ is a graphical function specifying the suicide attempt rate ratio for people becoming psychologically distressed as a result of the pandemic at time $t$ years since 1 March 2020 (the default function, shown in figure S23, is based on Milner et al., 2012).


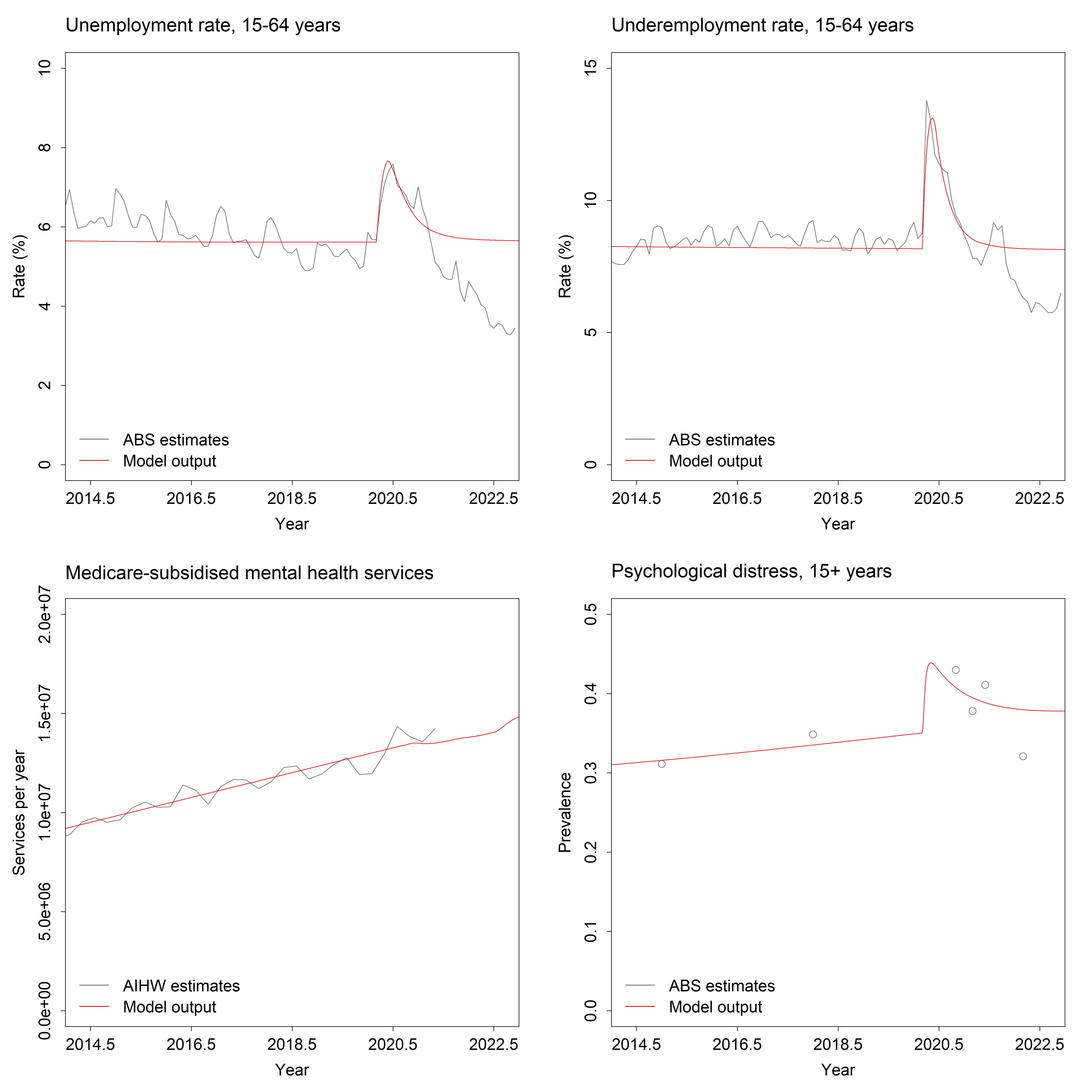


Figure S22. Modelled effects of the COVID-19 pandemic on unemployment, underemployment, mental health services usage, and psychological distress (red lines). Data published by the Australian Bureau of Statistics (ABS; 2022a, b) and the Australian Institute of Health and Welfare (AIHW; 2023) used in fitting the model are shown in grey.

References

Atkinson, J., Song, Y. J. C., Merikangas, K. R., Skinner, A., Prodan, A., Iorfino, F., Freebairn, L., Rose, D., Ho, N., Crouse, J., Zipunnikov, V., Hickie, I. B., 2020. The science of complex systems is needed to ameliorate the impacts of COVID-19 on mental health. Front. Psychiatry 11, 606035.


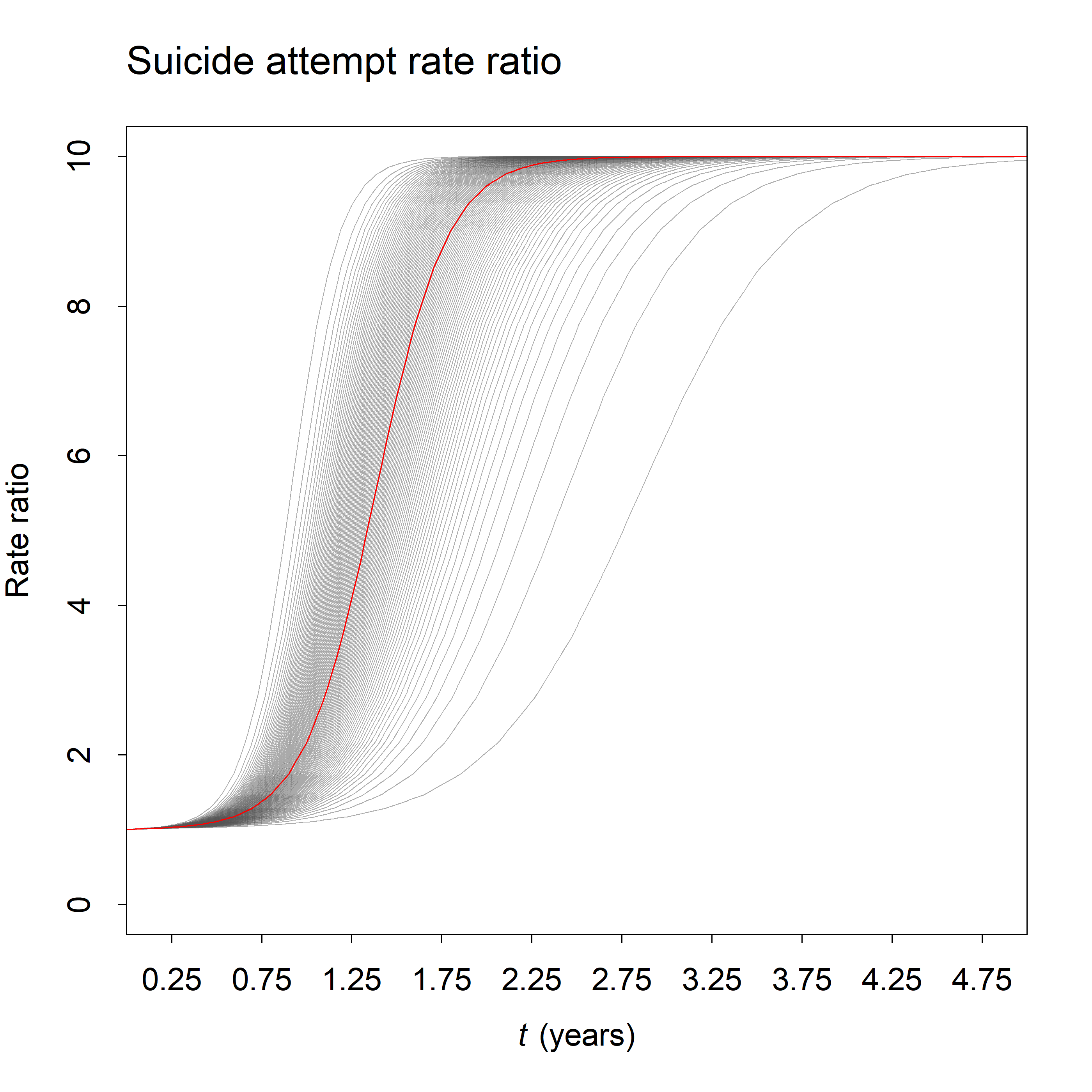


Figure S23. Graphical functions specifying the suicide attempt rate ratio for people becoming psychologically distressed as a result of the COVID-19 pandemic at time $t$ years since 1 March 2020. The grey lines are from the sensitivity analysis and were generated by multiplying the input time to the default function, shown in red, by a normal random variable with mean 1 and standard deviation 0.2, effectively compressing or stretching the function along the horizontal axis.

Australian Bureau of Statistics, 2022a. Labour force, Australia, Dec 2022. Cat. no. 6202.0. Australian Bureau of Statistics, Canberra.

Australian Bureau of Statistics, 2022b. Household Impacts of COVID-19 Survey, Mar 2022. Cat. no. 4940.0. Australian Bureau of Statistics, Canberra.

Australian Institute of Health and Welfare, 2023. Mental health. Medicare-subsidised mental health-specific services 2021−22. Available at: https://www.aihw.gov.au/mental-health/topic-areas/medicare-subsidised-services.

Biddle, N., Edwards, B., Gray, M., Sollis, K., 2020. Tracking outcomes during the COVID-19 pandemic (August 2020) — divergence within Australia. Centre for Social Research and Methods, Australian National University, Canberra.

Biddle, N., Gray, M., 2021. Tracking outcomes during the COVID-19 pandemic (January 2021) — cautious optimism. Centre for Social Research and Methods, Australian National University, Canberra.

Brooks, S. K., Webster, R. K., Smith, L. E., Woodland, L., Wessely, S., Greenberg, N., Rubin, G. J., 2020. The psychological impact of quarantine and how to reduce it: rapid review of the evidence. Lancet 395, 912−920.

Charles-Edwards, E., Wilson, T., Bernard, A., Wohland, P., 2020. How will COVID-19 impact Australia’s future population? A scenario approach. Working paper no. 2020/03. Queensland Centre for Population Research, University of Queensland, Brisbane.

Dooley, D., Catalano, R., Rook, K. S., 1988. Personal and aggregate unemployment and psychological symptoms. J. Soc. Issues 44, 107−123.

Garcy, A. M., Vågerö, D., 2013. Unemployment and suicide during and after a deep recession: a longitudinal study of 3.4 million Swedish men and women. Am. J. Public Health 103, 1031−1038.

Milner, A., Page, A., LaMontagne, A. D., 2013. Long-term unemployment and suicide: a systematic review and meta-analysis. PLoS ONE 8, e51333.

Moreno, C., Wykes, T., Galderisi, S., Nordentoft, M., Crossley, N., Jones, N., Cannon, M., Correll, C. U., Byrne, L., Carr, S., Chen, E. Y. H., Gorwood, P., Johnson, S., Kärkkäinen, H., Krystal, J. H., Lee, J., Lieberman, J., López-Jaramillo, C., Männikkö, M., Phillips, M. R., Uchida, H., Vieta, E., Vita, A., Arango, C., 2020. How mental health care should change as a consequence of the COVID-19 pandemic. Lancet Psychiatry 7, 813−824.

Supplementary appendix 3

Intervention definitions and parameter assumptions

Table S1. Interventions examined in the analyses.

| Intervention | Description |
| --- | --- |
|  |  |
| a. Mental health services capacity | Doubling of the annual rate of growth in community-based mental health services capacity (i.e., the total number of services that can be provided per week) from 2021. Community-based mental health services include Medicare-subsidised psychiatrist and allied mental health services and publicly funded hospital outpatient services. Current services capacity growth rates were estimated using data for the period 2011–2019 published by the Australian Institute of Health and Welfare (available at: https://www.aihw.gov.au/reports-data/health-welfare-services/mental-health-services/data). |
| b. Mental health awareness campaigns | Population-wide mental health education programs aimed at reducing stigma, improving recognition of suicide risk, and encouraging help-seeking. This intervention increases the per capita rates at which people perceive a need for mental health services and seek help from a general practitioner or online services. Parameters that can be modified include: *Starting year* — the year in which mental health awareness campaigns commence (the default is 2021). *Implementation time (years)* — the time after commencement required for mental health awareness campaigns to be fully implemented (the default is 0.167 years, or 2 months). *Program duration (years)* — the duration of mental health awareness campaigns (the default is 5 years). *Effect on engagement* — the multiplicative effect of mental health awareness campaigns on the per capita rates that people perceive a need for mental health care, seek help from a general practitioner, or access online services. The default value (1.585) is derived from Jorm et al. (2003, Psychol. Med. 33, 1071-1079). *Effect decay rate per year* — the fractional rate per year at which the effect on engagement decreases to a value of 1 (i.e., no effect) after mental health awareness campaigns end. The default value (1) implies that the effect on engagement would decrease to a value of 1 in 1 year given the initial rate of decline (i.e., the rate immediately after awareness campaigns end). Note that the rate of decline in the effect of awareness campaigns itself declines as the effect approaches a value of 1, so the actual time required for the effect to decay completely will generally be greater than the inverse of the decay rate specified. |
| c. Online services expansion | Increased investment in online (self-help) services providing support to people with relatively low care needs. This intervention increases the per capita rate that people with a perceived need for mental health care access online services. Parameters that can be modified include: *Starting year* — the year in which increased investment in online services commences (the default is 2021). *Implementation time (years)* — the time required to scale up investment in online services (the default is 0.167 years, or 2 months). *Program duration (years)* — the duration of increased investment in online services (the default is 5 years). *Increase in services usage* — the multiplicative effect of increased investment in online services on the rate that people perceiving a need for mental health care access those services. The default value (1.2) assumes that increased investment will increase the per capita rate of access to online services by 20%. |
| d. Post-suicide attempt care | Post-attempt care is an active outreach and enhanced contact program designed to reduce readmissions among those presenting to services after a suicide attempt. This intervention directly reduces the number of suicide attempts among people experiencing moderate to very high psychological distress. Parameters that can be modified include: *Starting year* — the year in which post-attempt care programs commence (the default is 2021). *Implementation time (years)* — the time after commencement required for post-attempt care programs to be fully implemented (the default is 2 years). *Program duration* — the duration of post-attempt care programs (the default is set to 1000 years, ensuring that programs remain in place until the end of the simulation). *Maximum rate* — the maximum proportion of patients hospitalised for a suicide attempt receiving post-attempt care. The default value (0.7) assumes that post-attempt care will be provided to 70% of patients hospitalised for a suicide attempt when post-attempt care programs are fully implemented. *Post-attempt care effect* — the proportion of potential repeat suicide attempts expected among patients receiving post-attempt care. The default value (0.398) implies that 39.8% of repeat attempts that would have occurred without post-attempt care actually occur when post-attempt care is provided; i.e., post-attempt care prevents 60.2% of potential repeat suicide attempts. The default estimate is derived from Hvid et al. (2011, Nord. J. Psychiatry 65, 292-298). *Repeat attempt rate per year* — the probability that a person will make a repeat suicide attempt in the year after a suicide attempt without post-attempt care. The default value (0.179) implies that 17.9% of people hospitalised for a suicide attempt will re-attempt within 1 year (i.e., assuming they do not receive post-attempt care); this estimate is derived from Carroll et al. (2014, PLoS ONE 9, e89944). |
| e. Technology-enabled coordinated care | Technology-enabled coordinated care involves the use of online technology to facilitate delivery of multidisciplinary team-based care, in which medical and allied health professionals consider all relevant treatment options and collaboratively develop an individual treatment and care plan for each patient. Online technology improves coordination of care and facilitates communication between medical and allied health professionals, as each health professional involved in the care of a patient has access to the same information about that patient’s treatment history. Parameters that can be modified include: *Starting year* — the year in which technology-enabled, measurement-based care is introduced (the default is 2021). *Implementation time (years)* — the time required for technology-enabled, measurement-based care to be fully implemented (the default is 2 years). *Program duration* — the duration of investment in technology-enabled, measurement-based care (the default is set to 1000 years, ensuring that investment continues until the end of the simulation). *Maximum rate per service* — the maximum proportion of mental health services provided that involve technology-enabled, measurement-based care. This proportion will depend on the number of medical and allied health professionals adopting online care coordination technologies, as well as the number of patients consenting to the use of these technologies in the management of their care (i.e., take-up among service providers and patients). The default value (0.7) assumes that technology-enabled, measurement-based care will be provided in 70% of mental health services completed when fully implemented. *Effect on recovery rate* — the multiplicative effect of technology-enabled coordinated care on the per-service recovery rate (i.e., the probability that a patient’s level of psychological distress will decrease after receiving treatment). The default estimate (1.177) is derived from Woltmann et al. (2012, Am. J. Psychiatry 169, 790-804), and implies that technology-enabled coordinated care increases the per-service probability of a reduction in psychological distress by 17.7%. *Effect on referral rate* — the multiplicative effect of technology-enabled, measurement-based care on general practitioners’ rates of referral to specialised mental health services (psychiatrists and allied mental health services). The default value (1.266) implies that technology-enabled, measurement-based care increases the per-consultation probability that a general practitioner will refer a patient with moderate to very high psychological distress to specialised psychiatric care by 26.6%, and is derived from Badamgarav et al. (2003, Am. J. Psychiatry 160, 2080-2090). *Effect on disengagement* — the multiplicative effect of technology-enabled, measurement-based care on per capita rates of disengagement from mental health services (including disengagement while waiting for services and disengagement resulting from dissatisfaction with services received). The default estimate (0.520) is derived from Badamgarav et al. (2003, Am. J. Psychiatry 160, 2080-2090), and implies that technology-enabled, measurement-based care reduces rates of disengagement by 48.0%. |
|  |  |

Supplementary appendix 4

Simulation results for adolescents and young adults (15−24-year-olds)


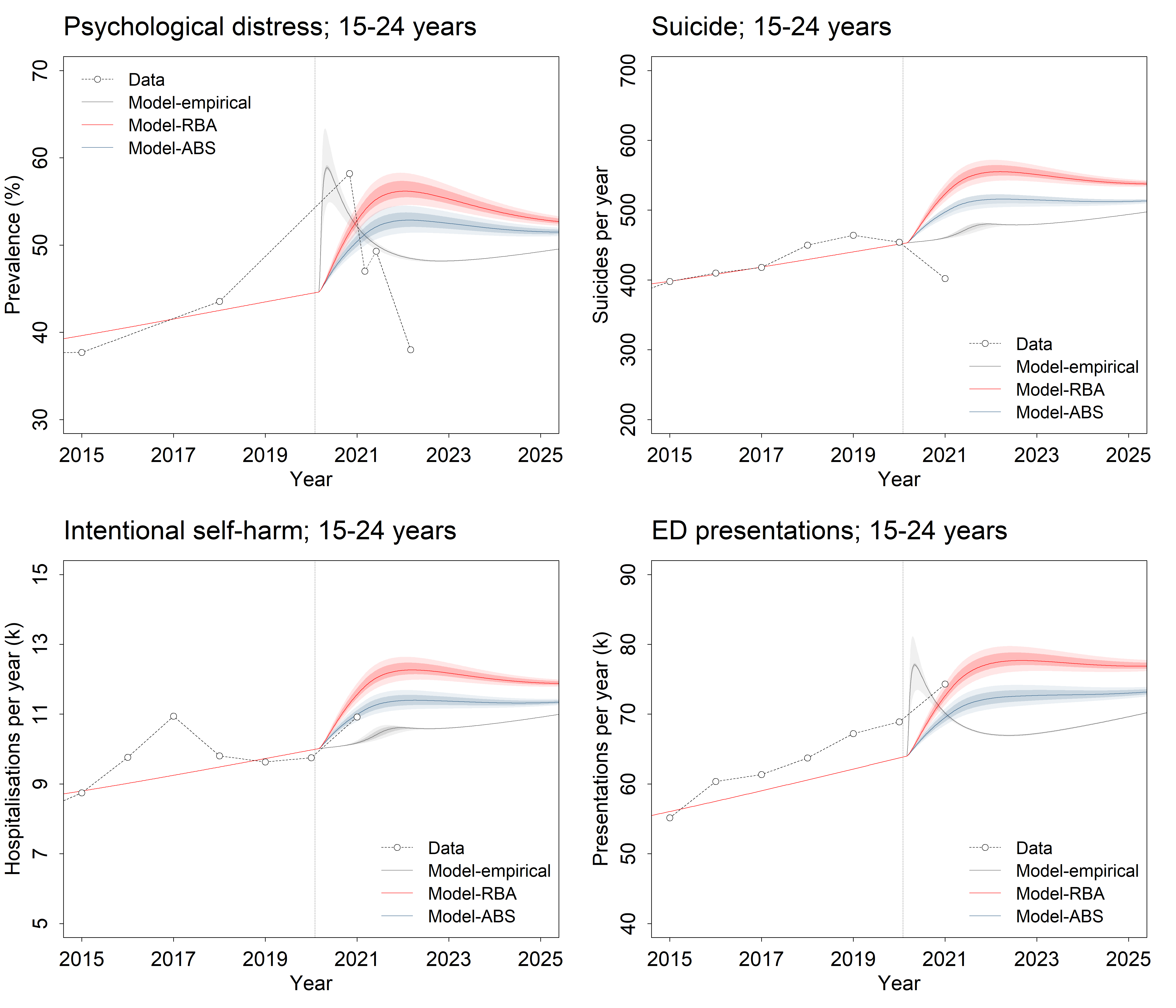


Figure S24. Simulation results for adolescents and young adults (15−24-year-olds). Pointwise 50% and 95% intervals derived from the sensitivity analyses are indicated with dark and light shading, respectively. The vertical dotted line in each plot indicates the start of the COVID-19 pandemic (30 January 2020). See Figure 2 of the paper for additional details.
